# New insights into the role of *Cutibacterium acnes*-derived extracellular vesicles in inflammatory skin disorders

**DOI:** 10.1038/s41598-023-43354-w

**Published:** 2023-09-25

**Authors:** Maria Pol Cros, Júlia Mir-Pedrol, Lorena Toloza, Nastassia Knödlseder, Julien Maruotti, Christos C. Zouboulis, Marc Güell, Maria-José Fábrega

**Affiliations:** 1 Department of Medicine and Life Sciences, Universitat Pompeu Fabra, Barcelona, Spain; 2 Quantitative Biology Center, University of Tuebingen, Tuebingen, Baden-Württemberg Germany; 3 Phenocell, Grasse, France; 4 Hochschulklinik für Dermatologie, Venerologie und Allergologie, Immunologisches Zentrum, Städtisches Klinikum Dessau, Medizinische Hochschule Brandenburg Theodor Fontane und Fakaltät für Gesundheitswissenschaften Brandenburg, Auenweg, Germany

**Keywords:** Biotechnology, Immunology, Microbiology, Molecular biology, Diseases, Health care

## Abstract

*Cutibacterium acnes* (*C. acnes*) is one of the most prevalent bacteria that forms the human skin microbiota. Specific phylotypes of *C. acnes* have been associated with the development of *acne vulgaris*, while other phylotypes have been linked to healthy skin. In this scenario, bacterial extracellular vesicles (EVs) play a role in the interkingdom communication with the human host. The purpose of this study was to examine the impact of EVs generated by various phylotypes of *C. acnes* on inflammation and sebum production using different in vitro skin cell types. The main findings of this study reveal that the proteomic profile of the cargo embodied in the EVs reflects distinct characteristics of the different *C. acnes* phylotypes in terms of life cycle, survival, and virulence. The in vitro skin cell types showed an extended pro-inflammatory modulation of SLST A1 EVs consistently triggering the activation of the inflammation-related factors IL-8, IL-6, TNFα and GM-CSF, in comparison to SLST H1 and SLST H2. Additionally, an acne-prone skin model utilizing PCi-SEB and arachidonic acid as a sebum inducer, was employed to investigate the impact of *C. acnes* EVs on sebum regulation. Our findings indicated that all three types of EVs significantly inhibited sebum production after a 24-h treatment period, with SLST H1 EVs exhibiting the most pronounced inhibitory effect when compared to the positive control. The results of this study highlight the protective nature of *C. acnes* SLST H1 EVs and their potential use as a natural treatment option for alleviating symptoms associated with inflammation and oily skin.

## Introduction

The human body harbours a flourishing diversity of microbes which form a dynamic and functional system that develops in synergy with the physiological development of its host^1^. The human microbiome resides primarily in the skin, the oral mucosa, the gastrointestinal tracts and the vagina^2–4^. Recent data indicates that the specific composition of the microbial community is associated with health and disease, which suggests that a detailed characterization of the microbial community would reveal important commensal host-microbiome as well as microbe-microbe interactions with therapeutic implications^5^. Over the past decade, researchers have discovered evidence of extensive communication between bacteria, skin cells and immune cells. These interactions lead to the strengthening and reparation of the barrier formed by the skin, which can reinforce the body’s defences against infection, and reduce excess inflammation^6^. Nonetheless, if there is a failure in the interkingdom communication, this protective function of the skin can be disrupted and results in dysbiosis between commensals and pathogens.

One of the most common bacterium present in the normal human skin microbiota is the Gram-positive anaerobic bacterium, *Cutibacterium acnes* (*C. acnes*) which dominate the upper 1.2–1.9 mm of terminal of pilosebaceous units^7–9^. *Cutibacterium acnes* help to preserve and support the natural microbial balance of the skin, however, under certain conditions it can also substantially alter its local environment and cause disease^10–12^. In fact, this bacterium is linked to a wide range of skin diseases, including *acne vulgaris*. Although the aetiology and pathogenesis of *acne vulgaris* are still uncertain, microbiome alterations are associated with its development^3^. More recent evidence leads to the hypothesis that different phylotypes of *C. acnes* might have different degrees of association with *acne vulgaris*^8,13–15^. This poses the question how bacteria may communicate with host cells to maintain homeostasis of the skin microbiota if sebaceous glands and immune cells are protected by a layer of sebum from an external environment. It has been demonstrated that under certain conditions *C. acnes* is also able to pass through the sebum layer and access the sebaceous glands^16^. Nonetheless, the main communication is known to be mainly mediated by bacterial extracellular vesicles (EVs), which are nano-sized lipid bilayer vesicles approximately 20–200 nm in diameter that can easily pass through the lipid barrier and internalize within host cells^17,18^.

Since EVs play a key role in interkingdom communication^19^ and immune modulation^20^, different in vitro cell cultures mimicking human skin have been established to assay the potential of different *C. acnes* EVs in the present study. In this context and based on previous publications^3,21,22^ we decided to study the role that EVs from three different phylotypes of *C. acnes* play in the onset of *acne vulgaris*. *Cutibacterium acnes* SLST A1 (phylotype IA1) can be considered as an acne-prone phylotype, whereas *C. acnes* SLST H1 (phylotype IB) and *C. acnes* SLST H2 (phylotype IB) can be considered as protective commensals. Moreover, proteomic characterization of *C. acnes* SLST A1, H1 and H2 EVs has been carried out to determine the relationship and influence of their protein cargo with the skin microbiota. Finally, a novel model mimicking acne-prone skin has been set up in which the sebum-reducing potential of EVs from different *C. acnes* phylotypes has been tested.

## Materials and methods

### Bacterial phylotypes and culture conditions

*Cutibacterium acnes* phylotypes used in this study were extracted from human skin (A1 and H1) or commercially acquired (H2, KPA17202 from DSMZ), typed by SLST typing and we previously sequenced. Sequencing data can be found in the European nucleotide Archive under project number PRJEB42527.

Initial cultures were always started from glycerol stocks (stored at − 80 °C) and seeded on Brucella agar plates which were incubated for 4–5 days at 37 °C under anaerobic conditions using the BD GasPak EZ anaerobic bag system.

### Isolation of EVs

The process followed for the isolation of EVs consisted of several steps: cultivation, centrifugation, concentration or ultrafiltration and ultracentrifugation.

In the cultivation step, *C. acnes* phylotypes SLST A1, H1 and H2 were collected from a Brucella agar plate using sterile swabs (Deltalab) and were transferred into 1 L of Brucella liquid medium. After 7–10 days at 37 °C with 300 rpm of stirring and using the BD GasPak EZ bag system to maintain anaerobic conditions, the culture was centrifuged in 250 mL sterile bottles at 10,000 × g for 30 min at 4 °C. For this step, a Beckman Coulter Avanti JXN-26 high-velocity centrifuge with the J-LITE JLA-16.250 Fixed-Angle Aluminium Rotor were used. Then, the pellet was discarded, and the supernatant (SN) was filtered using the Thermo Scientific Nalgene Rapid-Flow 0.2 μm Filter Unit (500 mL) to avoid bacterial contamination. Afterwards, the SN was concentrated by a Centricon Plus-70 10 KDa system using the JS-5.3 Swinging-Bucket Rotor. To ensure free contamination during the process, an extra step of 0.22 μm filtration was added to the protocol. The filtered and concentrated SN was then ultra-centrifuged in a Beckman Coulter optima L-100 XP ultracentrifuge using the SW41 Ti Swinging-Bucket Rotor at 154,300 × g for 2 h at 4 °C to precipitate the EVs. The pellet containing the isolated *C. acnes* EVs was washed with 1X phosphate buffered saline (PBS) buffer and ultra-centrifuged again for 1 h under the same conditions as in the previous step. Finally, the SN was discarded, and the pellet was resuspended with 200 μL of PBS buffer and kept in an Eppendorf at − 20 °C. To ensure non-bacterial presence in EVs samples, 10 μL of each batch were seeded in a Brucella agar plate and incubated for 4–5 days at 37 °C under anaerobic conditions using the BD GasPak EZ bag system.

### A1 EVs labelling protocol

EVs labelling was performed as previously described^17,23,24^. Briefly, isolated SLST A1 EVs were resuspended in PBS buffer, centrifuged at 154,300 × g for 2 h at 4 °C, and resuspended in labelling buffer (50 mM Na_2_CO_3_, 100 mM NaCl, pH 9.2) in the presence of 1 mg/mL of octadecyl rhodamine B-R18 (Invitrogen) and incubated for 1 h at 25 °C. Labelled SLST A1 EVs were pelleted by ultracentrifugation at 154,300 × g for 1 h at 4 °C, resuspended in PBS buffer (0.2 M NaCl) and washed three times to fully remove the unbound dye. After the final ultracentrifugation step, B-R18 labelled A1 EVs were resuspended in PBS buffer (0.2 M NaCl) containing a protease inhibitory cocktail (Complete Protease Inhibitory Tablets, Roche) and were stored at − 20 °C.

### Negative staining and transmission electron microscopy (TEM)

Isolated *C. acnes* SLST H1 and A1 EVs were examined by TEM after negative staining, as described in previous publications^25^. In this case, EVs were resuspended in TRIS buffer as PBS buffer is not compatible with uranyl acetate. A drop of EVs suspension was absorbed for 2 min on Formvar/carbon-coated grids that were previously activated with UV light. The grids were washed with distilled water, stained with 2% uranyl acetate for 1 min, air dried and examined by TEM (*Parc Científic de Barcelona*).

### Nanoparticle tracking analysis (NTA) of EVs

EVs resuspended in 200 μL of PBS buffer were sent to *Institut de Ciència de materials de Barcelona* to be examined with the Malvern Nanosight NS300 instrument. This system uses NTA technology characterize nanoparticles in suspension in the size range of 10–2000 nm. A video is taken and the NTA software tracks the Brownian motion of individual EVs and calculates their size and total concentration.

### Determination of protein concentration and SDS-PAGE

Protein concentrations in EVs samples were determined using the Qubit Protein Assay Kit (Thermo Fisher Scientific). Measuring was done using the Qubit Fluorometer (Thermo Fisher Scientific). This assay was performed at room temperature using specific Qubit assay tubes (Cat. no. Q32856).

For protein separation, the samples were subjected to sodium dodecyl sulfate-polyacrylamide gel electrophoresis (SDS-PAGE) using the Mini Gel Tank electrophoresis system (Invitrogen). Proteins were denatured in a loading buffer (1X LDS sample buffer + β -mercaptoethanol) at 98 °C for 5 min. Samples were loaded and separated on a NuPAGE 4–12% Bis–Tris Gel (Invitrogen) using 1X MOPS SDS Running Buffer 20X (Life technologies) at a constant voltage of 110 V and for 1:30 min. After the run was completed, several bands were identified. Gel images were visualized in the ChemiDoc MP Imaging System (Bio-Rad).

### Proteomic analysis: EVs preparation

EVs were lysed to get a total representation of their protein content. To do that, liquid nitrogen (N_2_) was used to obtain beads of the EVs solutions of each *C. acnes* phylotype. Then, 0.5 g of beads were disrupted using the Freezer Mill. Conditions used for the lysis were: precooling for 5 min, running step for 2 min, 2 cycles of interval and at a rate of 10 cycles per second (CPS). The powder obtained was resuspended in RIPA buffer supplemented with complete protease inhibitory tablets (Roche). Afterwards, total protein was precipitated with acetone and the final precipitate was resuspended with a quantity ranging from 50 to 250 μL of 6 M urea to have a minimum of 8 μg of protein. Qubit protein assay was performed to determine the estimated quantity of protein in each EV sample.

### Identification of EVs proteins by LC–MS/MS analysis

For the identification of the EVs proteins, mass spectrometry was performed by the UPF/CRG Proteomics Unit.

Samples (8 μg) were reduced with dithiothreitol (24 nmol, 37 °C, 60 min) and alkylated in the dark with iodoacetamide (48 nmol, 25 °C, 30 min). The resulting protein extract was first diluted to 2 M urea with 200 mM ammonium bicarbonate for digestion with endoproteinase Lys-C (1:10 w:w, 37 °C, o/n, Wako, cat # 129-02541), and then diluted twofold with 200 mM ammonium bicarbonate for trypsin digestion (1:10 w:w, 37 °C, 8 h, Promega cat # V5113). After digestion, the peptide mix was acidified with formic acid and desalted with a MicroSpin C18 column (The Nest Group, Inc) prior to LC–MS/MS analysis.

Afterwards, samples were analysed using the LTQ—Orbitrap Fusion Lumos mass spectrometer (Thermo Fisher Scientific, San Jose, CA, USA) coupled with an EASY-nLC 1200 (Thermo Fisher Scientific (Proxeon), Odense, Denmark). Peptides were loaded directly onto the analytical column and were separated by reversed-phase chromatography using a 50-cm column with an inner diameter of 75 μm, packed with a 2 μm C18 particles spectrometer (Thermo Scientific).

Chromatographic gradients started at 95% buffer A and 5% buffer B with a flow rate of 300 nL/min for 5 min and gradually increased to 25% buffer B and 75% A in 79 min and then to 40% buffer B and 60% A in 11 min. After each analysis, the column was washed for 10 min with 10% buffer A and 90% buffer B. Buffer A: 0.1% formic acid in water. Buffer B: 0.1% formic acid in 80% acetonitrile.

The mass spectrometer was operated in positive ionization mode with nanospray voltage set at 2.4 kV and source temperature at 305 °C. The acquisition was performed in data-dependent acquisition (DDA) mode and full MS scans with 1 micro scan at a resolution of 120,000 were used over a mass range of m/z 350–1400 with detection in the Orbitrap mass analyser, auto gain control (AGC) was set to “Standard” and maximum injection time to “Auto”. In each cycle of data-dependent acquisition analysis, following each survey scan, the most intense ions above a threshold ion count of 10,000 were selected for fragmentation. The number of selected precursor ions for fragmentation was determined by the “Top Speed” acquisition algorithm and a dynamic exclusion of 60 s. Fragment ion spectra were produced via high-energy collision dissociation (HCD) at a normalized collision energy of 28% and they were acquired in the ion trap mass analyser. AGC was set to 2E4, and an isolation window of 0.7 m/z and a maximum injection time of 12 ms were used. Digested bovine serum albumin (New England Biolabs cat # P8108S) was analysed between each sample to avoid sample carryover and to assure stability of the instrument and QCloud^26^ has been used to control instrument longitudinal performance during the project.

Acquired spectra were analysed using the Proteome Discoverer software suite (v2.0, Thermo Fisher Scientific) and the Mascot search engine (v2.6, Matrix Science,^27^). The data were searched against NCBI *acnes* phylotype H2171202 database (as in August 2021, 2476 entries, https://www.ncbi.nlm.nih.gov/nuccore/NC_006085.1). For peptide identification a precursor ion mass tolerance of 7 ppm was used for MS1 level, trypsin was chosen as the enzyme, and up to three missed cleavages were allowed. The fragment ion mass tolerance was set to 0.5 Da for MS2 spectra. Oxidation of methionine and N-terminal protein acetylation were used as variable modifications whereas carbamidomethylation on cysteines was set as a fixed modification. The false discovery rate (FDR) in peptide identification was set to a maximum of 5%. Peptide quantification data were retrieved from the “Precursor ion area detector” node from Proteome Discoverer (v2.0) using 2 ppm mass tolerance for the peptide extracted ion current (XIC). The obtained values were used to calculate protein fold changes and their corresponding adjusted *p*-values.

### Bioinformatic analysis

Proteins found by mass spectrometry were annotated from the UniProt database (UniProt Consortium, 2021). Pseudo proteins or proteins lacking information in UniProt were removed from the analysis. Only proteins with an area found in two or three of the replicas were considered. The mean of the areas of each protein was calculated.

Proteins present only in one of the three phylotype EVs were selected for further analysis. The information on biological processes, cellular components and molecular functions was obtained and manually curated to classify each protein into a category.

### In vitro human cell lines maintenance

The immortalized keratinocytes cell line (HaCaT), bought in the American Type Culture Collection (ATCC), were cultured in T-75 culture flasks (Thermo Scientific) with Dulbecco’s Modified Eagle Medium (DMEM, Gibco) supplemented with 10% of Fetal bovine serum (FBS) and 0.1% of penicillin and streptomycin. They were incubated at 37 °C in 5% of CO_2_, and 95% of humidity until 90–100% of confluency.

Immortalized human SZ95 sebocytes^28^ were cultured in T-75 culture flasks (Thermo Scientific) with Sebomed basal medium (DMEM/F12, Gibco) supplemented with 10% of FBS, 0.1% of penicillin and streptomycin, 5 ng/mL of recombinant human epidermal growth factor (rEGF) and 500 μL of filtrated calcium chloride (CaCl_2_) 1 M. They were incubated at 37 °C in 5% of CO_2_, and 95% of humidity until 90–100% of confluency.

The immortalized T lymphocytes cell line (Jurkat, Clone E6-1), bought in the ATCC, were cultured in T-75 culture flasks (Thermo Scientific) with Roswell Park Memorial Institute medium (RPMI, Gibco) supplemented with 10% of FBS and 0.1% of penicillin and streptomycin. They were incubated at 37 °C in 5% of CO_2_, and 95% of humidity until 90–100% of confluency.

PCi-SEB, derived from human iPSC with a Caucasian phototype (Phenocell), were seeded at a density of 25.000 viable cells/cm^2^ on fibronectin-coated tissue culture plate (Falcon) in PhenoCULT-SEB basal medium supplemented with 0.1% of supplement A. Then, the media was changed and supplemented with 0.1% of supplement M and incubated for 2 days more until cells reached 90% of confluence and they were differentiated into mature sebocytes. Cells were incubated at 37 °C in 5% CO_2_ and 95% of humidity during the whole differentiation process.

### Internalization of labelled-EVs

To monitor EVs uptake by both HaCaT and SZ95 sebocytes, a total of 1 × 10^5^ cells were seeded in an 8-well chamber slider (Ibidi) until approximately 80% of confluence. Before the assay, the medium was aspirated and replaced with rhodamine B-R18-labelled SLST A1 EVs (2 μg/mL) suspended in DMEM and Sebomed both without fetal bovine serum and red phenol. The cells with the labelled SLST A1 EVs were incubated at 37 °C in 5% of CO_2_, and 95% of humidity for 24 h.

### Confocal fluorescence microscopy

Cells were fixed for 30 min with 3% of paraformaldehyde at room temperature (RT) and washed with PBS buffer to eliminate possible EVs residues. Plasma membranes were labelled with fluorescent Alexa-488 wheat germ agglutinin (WGA) (Invitrogen) and nuclei with Hoechst (Invitrogen). For this, cells were incubated for 25 min with Alexa-488 WGA (1 μg/mL) and during 10–15 min with Hoechst (3 drops/mL). After PBS buffer washing, Ibidi mounting medium was added (3 drops/well) to prepare the cells for microscopic visualisation.

Confocal microscopy was carried out using a ZEISS LSM 980 with Airyscan 2 confocal microscope, using the 63X oil immersion objective lens. Fluorescence was recorded at 405 nm (blue; Hoechst), 488 nm (green; WGA) and 546 nm (red; rhodamine B-R18). Z-stack images were taken at 1.0-μm. Images were analysed using the Fiji image processing package.

### Cytotoxicity assay

To evaluate cytotoxicity, an XTT Cell Viability Assay (Invitrogen) was performed following the manufacturer’s protocol. The assay kit includes the XTT reagent and an Electron Coupling Reagent. In this case, the XTT reagent, is sensitive to cellular redox potential and in the presence of live cells converts from a water-soluble compound to an orange-coloured formazan product.

2 × 10^5^ cells/mL of HaCaT and SZ95 sebocytes were seeded in two different 96-well plates. After 24 h, cells were incubated with 1.56 μg/mL, 3.125 μg/mL, 6.25 μg/mL, 12.5 μg/mL, 25 μg/mL, 50 μg/mL, 100 μg/mL, and 200 μg/mL of *C. acnes* A1, H1 and H2 EVs for 24 h at 37 °C in 5% of CO_2_ and 95% of humidity. Then, 70 μL of working solution (XTT reagent) were added directly to each well. Cells were incubated for 4 h at 37 °C in 5% of CO_2_ and 95% of humidity. After incubation, the absorbance was measured in the plate reader (Tecan) at 450 nm and 660 nm to eliminate the background signal contributed by cell debris or other non-specific absorbance. Negative controls with non-treated cells were included as blanks.

The percentage of cell viability was calculated using the following formula:

$$\text{\% Cell } \, \text{Viability} = \frac{{\text{A}}{\text{bsorbance}} \, \text{ in } \, {\text{Test}} \, \, {\text{well}}}{{\text{Absorbance}} \, \text{ in} \, \text{ Blank } \, {\text{well}}}{\text{x}}{100}$$

### *Cutibacterium acnes* EVs incubation in keratinocytes, sebocytes, and lymphocytes cell cultures

*Cutibacterium acnes* SLST A1, H1 and H2 EVs were incubated with the immortalized HaCaT, SZ95 sebocytes and Jurkat cell lines (Fig. S2). For this purpose, 2 × 10^5^ cells/mL were seeded in a 12-well plate for the three cell lines. After 24 h, all cell types were incubated for 24 h with *C. acnes* A1, H1 and H2 EVs. The three plates were divided into three rows—one for each *C. acnes* phylotype—and each row had a control well and different concentrations of EVs, namely 12.5 μg/mL, 25 μg/mL, and 50 μg/mL.

### Quantitative real time-PCR analysis (RT-qPCR)

Total RNA was extracted from HaCaT, SZ95 sebocytes and Jurkat cells using the miRNeasy Mini Kit (Qiagen) following the manufacturer’s recommendations. Purity and RNA concentration were measured by the absorbance ratio at 260 and 280 nm on the NanoDrop One spectrophotometer (Thermo Scientific).

RNA was reverse transcribed using the cDNA Reverse Transcription Kit (Thermo Fisher Scientific) in a final volume of 20 μL following the manufacturer’s protocol. The retro transcription reaction was performed in the ProFlex PCR System (Applied Biosystems). RT-qPCR reactions were performed on the QuantStudio 7 Flex Real-Time PCR System (Applied Biosystems) using SYBR Green PCR Master Mix (Applied Biosystems) and cell-type specific primers. See the list of primers in Table S4 of the Supporting Information. A control reaction was set up with water in which no RNA was present. The 2^-ΔΔCt^ method was used to normalise expression results. The values of the housekeeping CREBBP gene were used to standardise the values obtained for each of the genes being studied.

### Multiplex panel (Eve technologies)

To validate the results obtained in the RT-qPCR, a Human High Sensitivity Plex Discovery Assay was performed (Eve Technologies). The targets of interest were selected from an extensive list of analytes and the company allowed multiplexing the previously selected targets together in a single assay.

### Evaluation of sebum-reducing potential of *C. acnes* SLST A1, H1 and H2 EVs in the acne-mimicking skin model

For the acne-mimicking skin model, human iPSC-derived sebocytes (PCi-SEB) were incubated for 5 days with maturation supplement following manufacturer’s instructions. After that, the sebum production was induced by treating cells for 48 h of treatment with 5 μM of arachidonic acid (AA5). After 48 h, cells were treated with 12.5 μg/mL of *C. acnes* SLST A1, H1 and H2 EVs for 24 h and cells were fixed until sebum production determination. In parallel, non-treated cells were used as negative control. Finally, cells were fixed for sebum production determination with BODIPY stain. For lipid droplets detection, cells were stained with the fluorescent marker BODIPY 493/503 (Sigma Aldrich) following manufacture’s recommendations. The nucleus was stained with DAPI. Samples were analysed by confocal fluorescence microscopy.

### Statistical analysis

Statistical analyses were performed with GraphPad software. All tests were repeated independently at least three times in triplicate. The values for all measurements are presented as the mean ± standard deviation (SD). Differences between more than two groups were assessed using one-way or two-way ANOVA followed by Dunnett’s test. Data with a *p* value less than 0.05 and less than 0.001 were considered statistically significant and statistically highly significant, respectively.

### Consent for publish

All authors give their consent for publication of this work as a research article.

## Results

### Isolation and visualization of *C. acnes* EVs

**Figure 1:**
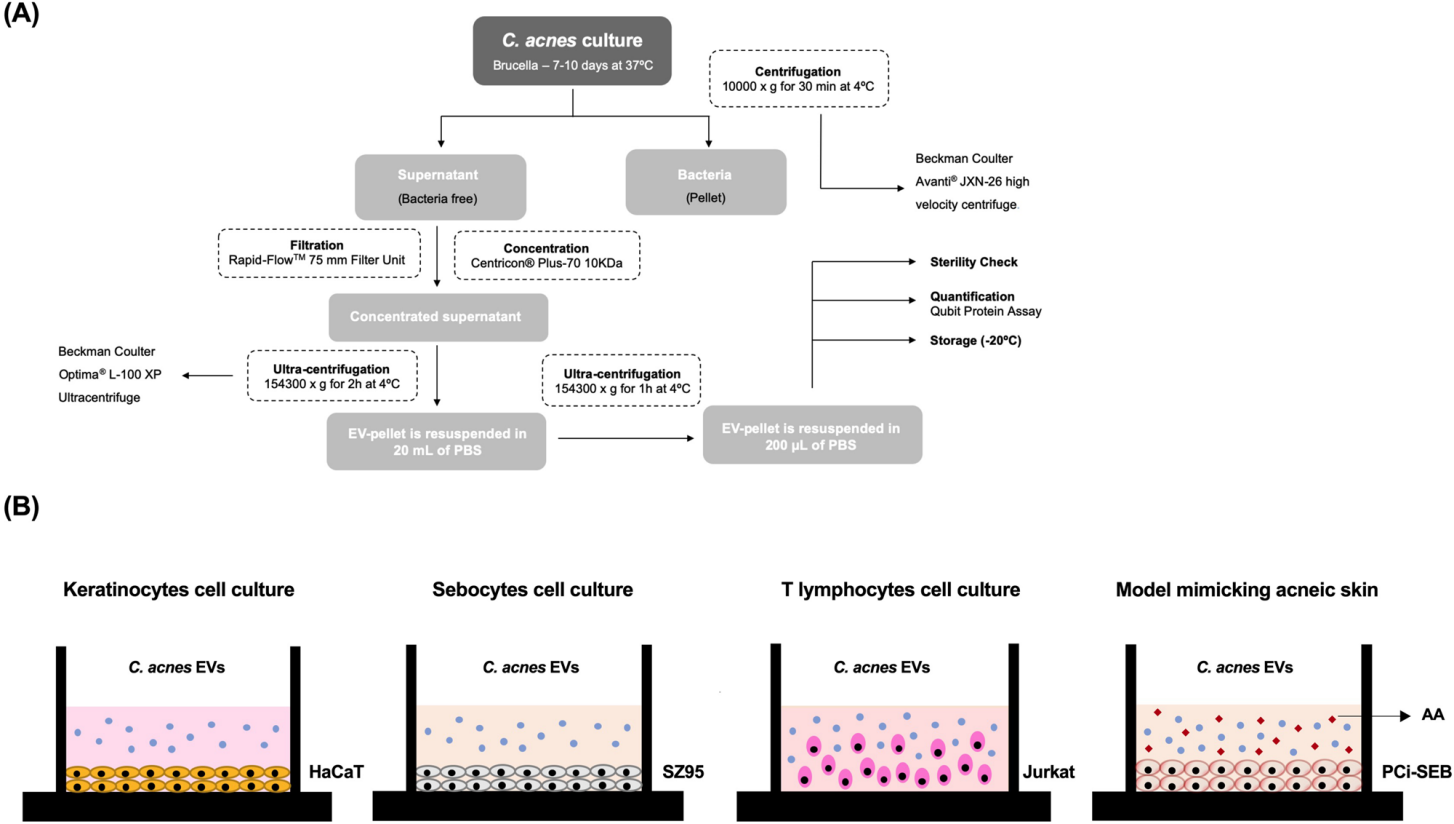
EVs isolation protocol and in vitro experimental design. (**A**) Process to isolate *C. acnes*-derived extracellular vesicles (EVs). (**B**) Schematic presentation of the different skin in vitro cell types: HaCaT cell line, SZ95 cell line, Jurkat cell line and the acne-prone skin model with PCi-SEB cells using AA to induce sebum production.

**Figure 2:**
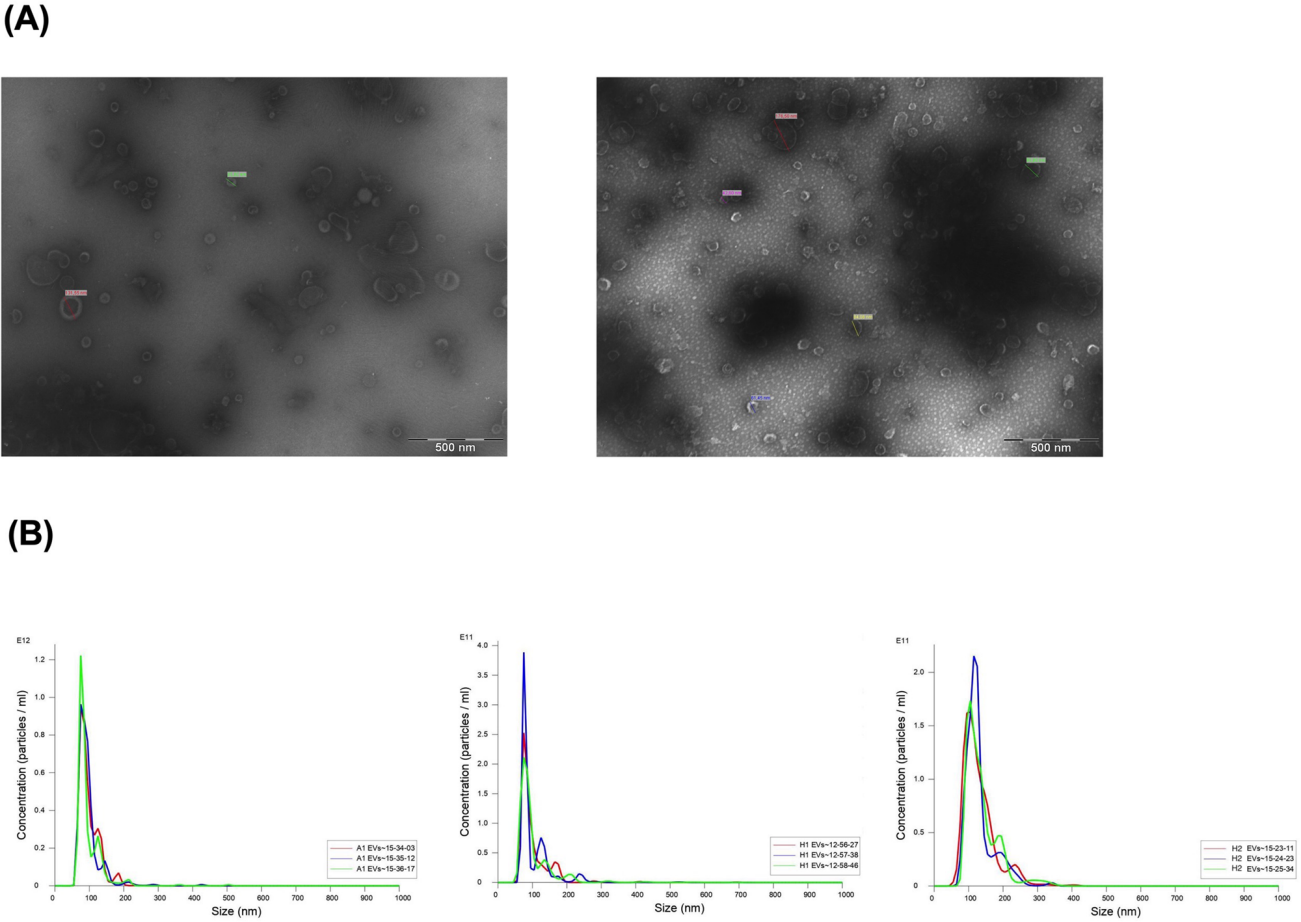
Visualization of *C. acnes* EVs by different microscopy techniques. (**A**) Transmission Electron Microscopy of *C. acnes* SLST H1 EVs and A1 EVs (bar 500 nm). (**B**) Nanoparticle Tracking Analysis of *C. acnes* SLST A1, H1 and H2 EVs. Prior to the measurement, the samples were diluted in PBS buffer at 1:5000, 1:1000 and 1:1000 respectively. EVs have a mean diameter of 96.8 ± 0.5 nm, 100.7 ± 0.7 nm and 133.0 ± 2.3 nm respectively. EV-concentration is expressed as a number of particles per mL on the y axis. All data represent the mean of three independent experiments ± standard error.

*Cutibacterium acnes* SLST H1 EVs and SLST A1 EVs were isolated from cell-free culture supernatants (Fig. 1) and evaluated by negative stain-TEM. The images show spherical vesicles measuring approximately 70–180 nm in diameter (Fig. 2A). To validate the TEM results and obtain additional information, NTA was conducted for *C. acnes* SLST A1, H1 and H2 EVs. NTA analysis offers high-resolution profiles of particle size distribution and accurate concentration measurements, calculating the secretion yield rate of *C. acnes* EVs. Figure 2B shows size distribution graphs for the different *C. acnes* EVs. The values obtained from the NTA analysis indicate that SLST H2 EVs have a larger mean vesicle size compared to SLST A1 and SLST H1 EVs. In terms of concentration, SLST A1 exhibits the highest EV secretion yield rate (3.92 × 10^12^ ± 1.31 × 10^11^ EVs/mL), followed by H2 (1.23 × 10^12^ ± 3.20 × 10^10^ EVs/mL) and H1(9.48 × 10^11^ ± 1.33 × 10^10^ EVs/mL). The NTA analysis also includes recordings of the different types of *C. acnes* EVs (see Videos).

In parallel, another faster EVs isolation methodology (EXOGAG from NasasBiotech) was considered for use with *C. acnes* EVs. However, it was observed that the polymeric nature of this reagent exhibited toxicity on keratinocytes cell cultures (Fig. S3). Consequently, this alternative EVs isolation methodology was discarded.

### Proteomic analysis comparison between *C. acnes* A1, H1 and H2 EVs

**Figure 3:**
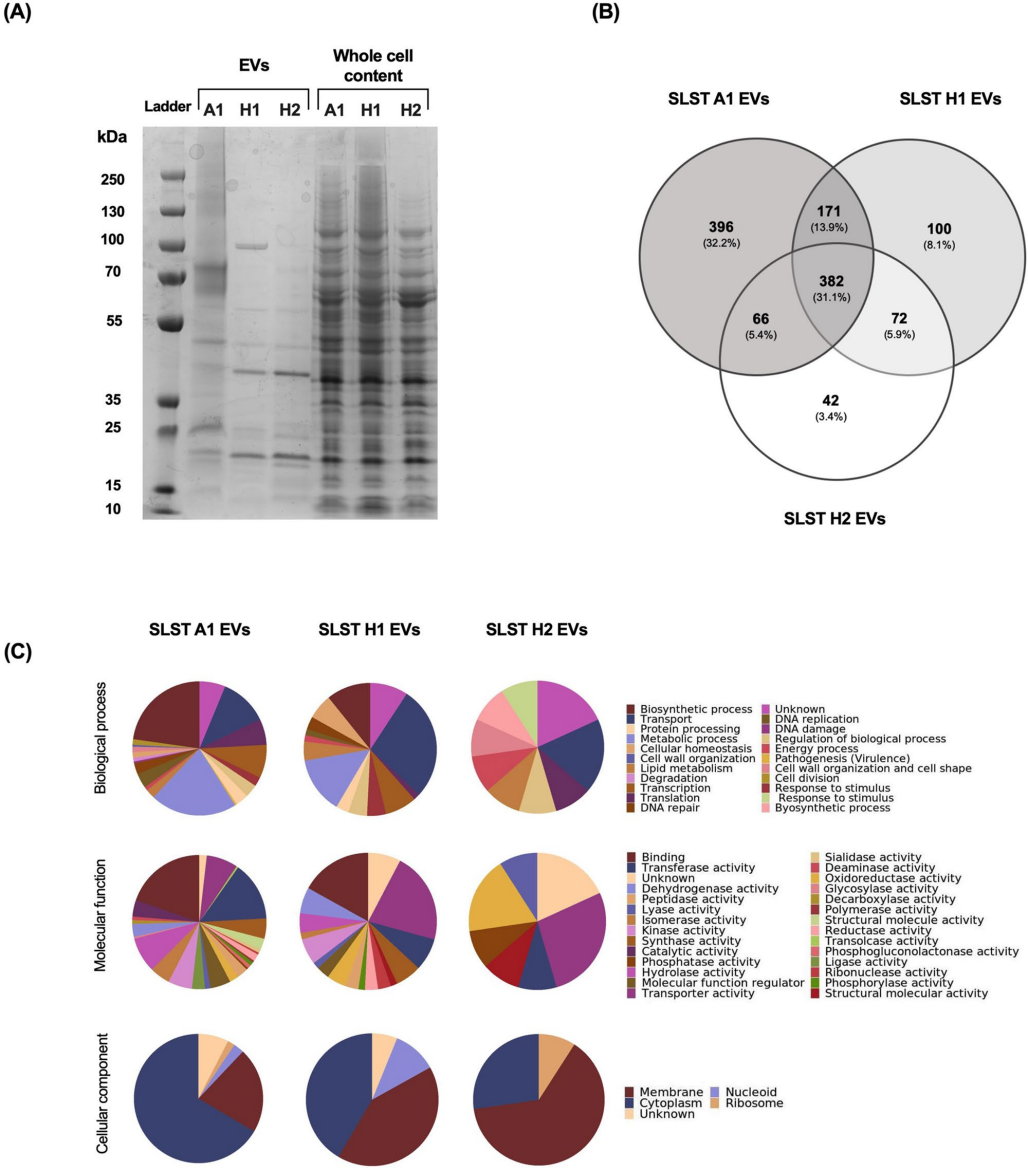
Proteomic characterization of *C. acnes* SLST A1, H1 and H2 EVs. (**A**) Proteomic fingerprint of *C*. *acnes* EVs and the corresponding protein whole cell content of each bacterium represented by an SDS-PAGE. (**B**) Venn diagram of the proteins contained in *C. acnes* SLST A1, H1 and H2 EVs. The number of overlapping proteins between the different phylotypes is indicated. All data is obtained from three independent experiments. (**C**) Gene Ontology analysis of *C. acnes* SLST A1, H1 and H2 EVs preparations. Biological process, Molecular function, and Cellular components of the identified vesicular proteins in *C. acnes* EVs are presented here. For each *C. acnes* phylotype, three independent batches were analysed in the proteomic analysis.

To undertake a preliminary characterisation of the *C. acnes* EVs, its proteomic profile was analysed by SDS-PAGE. As Figs. 3A and S4 show, a representative protein profile of each EVs phylotype (obtained from supernatant) and the corresponding whole protein fraction (obtained from bacterial pellet) was compared. Results revealed a different protein profile of EVs in comparison to the whole protein content of the bacterium. Furthermore, certain differences were observed between EVs types, aligning with recent findings presented by Chudzik et al.^22^.

To characterize the protein profile of *C. acnes* A1, H1 and H2 EVs, the protein content was isolated and examined by mass spectrometry. As Fig. 3B illustrates in a Venn diagram, the three phylotypes share 382 proteins. Additionally, a pairwise comparison was performed, and the results showed that A1 and H1 share 171 proteins, A1 and H2 share 66 proteins and H1 and H2 share 72 proteins.

Finally, observing the proteins exclusive to each phylotype it is shown that SLST A1 EVs have the highest number of high-confidence proteins identified, namely 396, followed by SLST H1 and H2 EVs.

Using the UniProt database, a Gene Ontology (GO) and bioinformatics analysis were conducted (Fig. 3C). In terms of biological processes, proteins participating in biosynthetic processes (22.77%) and metabolic processes (20.98%) were more abundant in *C. acnes* SLST A1 EVs. Regarding the molecular functions, binding (19.64%), transferase activity (14.29%) and hydrolase activity (8.48%) were the most prevalent. For biological processes, proteins related to transport (27.69%) and metabolic processes (13.85%) were found to be significantly more abundant in the *C. acnes* SLST H1 EVs. Regarding the molecular functions, transporter activity (21.54%) and binding (16.92%) were noteworthy. The predominant proteins involved in the biological processes of *C. acnes* SLST H2 EVs were linked to transport (18.18%) and response to stimulus (9.09%). Regarding the molecular functions of the vesicular proteins found in this phylotype, transporter activity (27.27%), and oxidoreductase activity (18.18%) were the most prevalent. For all three types of EVs, in terms of cellular components, the highest percentage of proteins were membrane-associated proteins, followed by cytoplasmic proteins. Also, approximately 6–18% of the total number of proteins were not properly classified as they were unknown or poorly characterized. The identified proteins in *C. acnes* SLST A1, H1 and H2 EVs are listed respectively in Tables S1, S2 and S3 of the Supporting Information.

### EVs from *C. acnes* SLST A1 phylotype are internalized into keratinocytes and sebocytes

**Figure 4:**
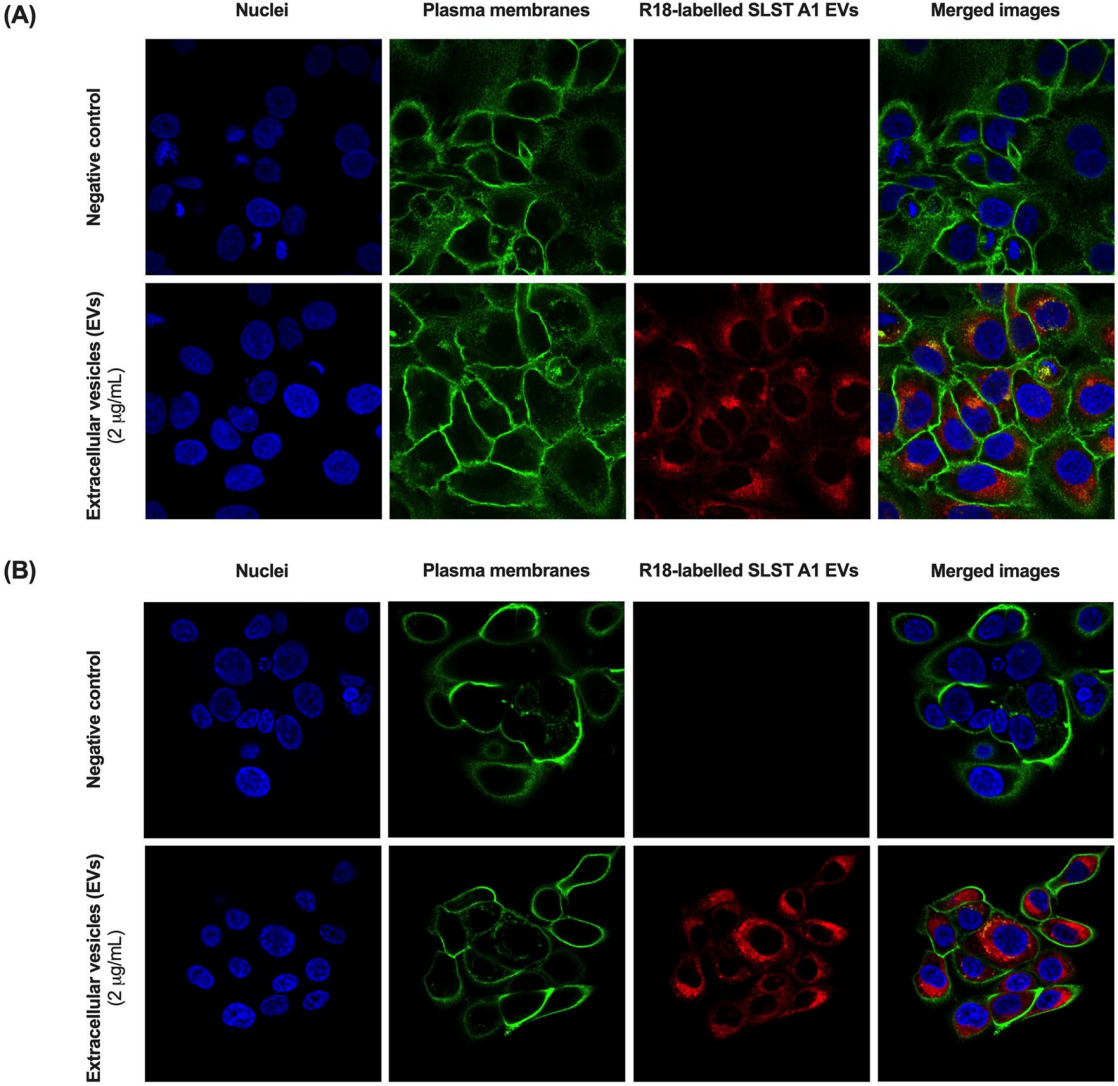
Uptake of *C. acnes* SLST A1 EVs in (**A**) HaCaT cell line and (**B**) SZ95 cell line. Visualization of internalized EVs by confocal fluorescence microscopy. Both cell lines were incubated for 24 h at 37 °C with rhodamine B-R18-labelled SLST A1 EVs (2 μg/ well).

*Cutibacterium acnes* EVs internalization in HaCaT keratinocytes and SZ95 sebocytes was confirmed by confocal fluorescence microscopy at 24 h of incubation with rhodamine B-R18-labeled SLST A1 EVs (2 μg/ well). Membranes were labelled with WGA and nuclei with Hoechst. SLST A1 EVs were found in the perinuclear area, indicating that EVs were endocytosed into cells. As anticipated, no red signal was observed in untreated control cells (Fig. 4).

### From a concentration of 50 μg/mL and below, *C. acnes* EVs do not appear to affect cell viability in the two different skin cell types tested

**Figure 5:**
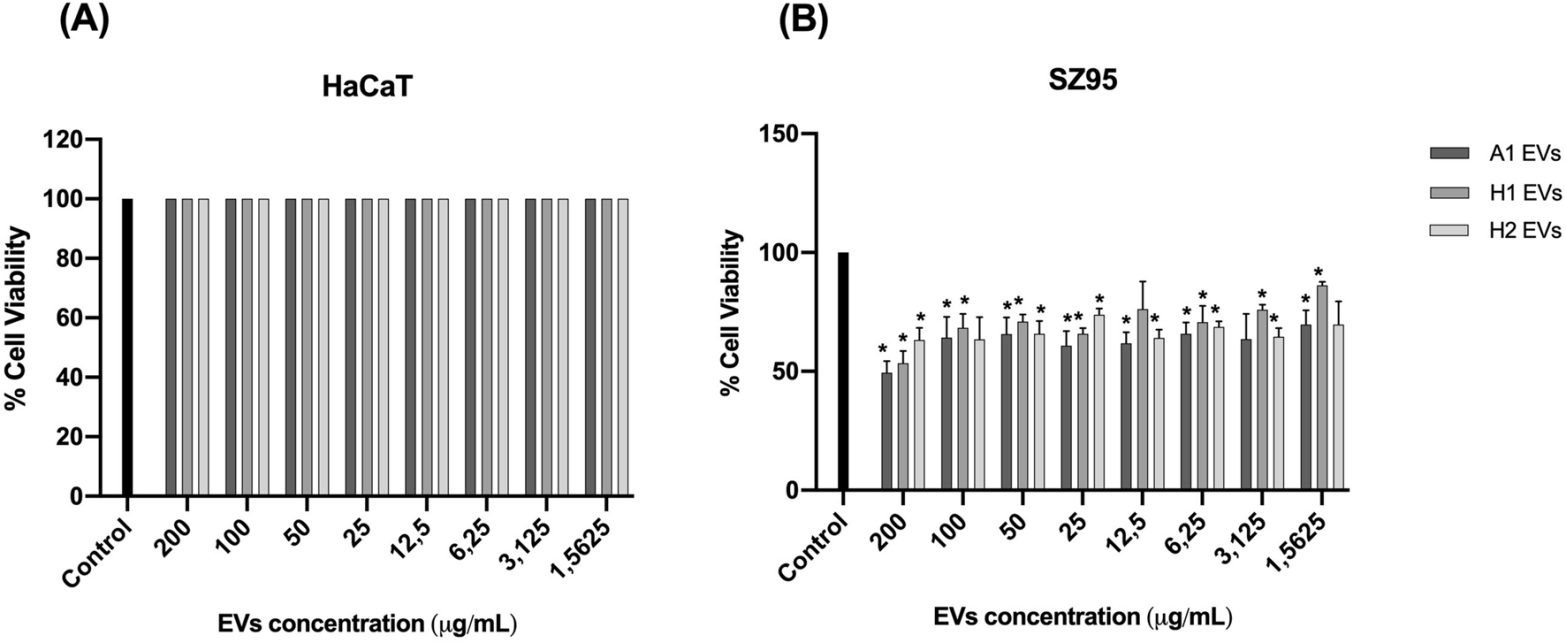
Cytotoxicity assay of (**A**) HaCaT cell line and (**B**) SZ95 sebocyte cell line treated with different doses of *C. acnes* SLST A1, H1 and H2 EVs. All data are presented as mean ± standard deviation (SD) of triplicate measurements (*p ≤ 0.05 vs. non-stimulated controls).

A cytotoxicity assay was performed in HaCaT and SZ95 cells. In both tests, *C. acnes* SLST A1, H1 and H2 EVs were added at different doses (1.56 μg/mL, 3.125 μg/mL, 6.25 μg/mL, 12.5 μg/mL, 25 μg/mL, 50 μg/mL, 100 μg/mL and 200 μg/mL). As is shown in Fig. 5A, in the HaCaT cell type, cell viability is not reduced in any condition.

On the other hand, in the SZ95 cell culture, cell viability is reduced to 50–70% when concentrations of 200 μg/mL and 100 μg/mL of EVs are added (Fig. 5B). Nonetheless, in this cell type, when adding concentrations of 50 μg/mL and below, viability is lowered by approximately 15–20% in SLST A1 and H2 EVs but is maintained nearly at 100% in SLST H1 EVs.

### EVs released by *C. acnes* SLST A1, H1 and H2 phylotypes modulate differently the expression of biomarkers related to the immune system, oxidative stress, skin barrier and sebum production

**Figure 6:**
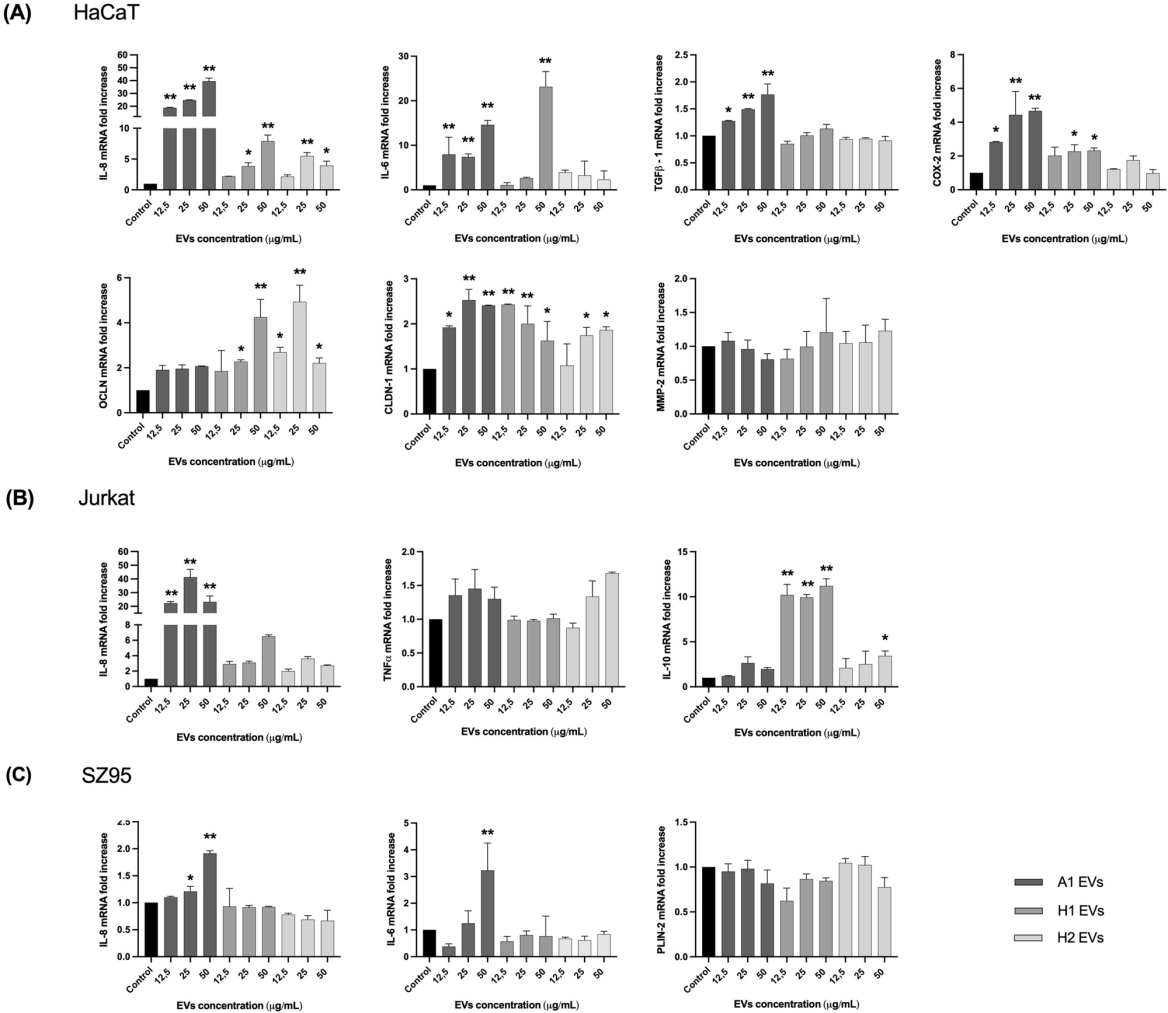
In vitro analysis with direct incubation of *C. acnes* EVs. Human cells were treated for 24 h with different concentrations (12.5, 25 and 50 μg/mL) of *C. acnes* SLST A1, H1 and H2 EVs. Total RNA was extracted, and different biomarkers were assessed by RT-qPCR. (**A**) HaCaT (**B**) SZ95 and (**C**) Jurkat immortalized cell lines. All data are presented as mean ± standard deviation (SD) of triplicate measurements (*p ≤ 0.05, **p ≤ 0.001 vs. non-stimulated controls).

Different in vitro skin cell types were used to evaluate the immunomodulatory properties of direct stimulation with a range of concentrations of *C. acnes* SLST A1, H1 and H2 EVs (Fig. 1B). In this sense, HaCaT, SZ95 and Jurkat immortalized cell lines were used^29–35^. All in vitro cell cultures were stimulated with *C. acnes* EVs for 24 h, and afterwards the expression level of different regulatory genes (immune system, skin barrier and oxidative stress) was measured by RT-qPCR. In Figs. 6A and S1A, RT-qPCR results show that SLST A1 EVs trigger a higher stimulation of the pro-inflammatory cytokines Interleukin-8 (IL-8), Interleukin-6 (IL-6) and transforming growth factor beta 1 (TGFβ-1), and the prostaglandin COX-2 which is linked to oxidative stress, compared to SLST H1 or H2 EVs. Regarding genes involved in the reinforcement of the skin barrier, such as occludin, all concentrations of SLST H2 EVs and high concentrations of SLST H1 EVs (25 μg/mL and 50 μg/mL) were the only ones able to induce a significant increase in the mRNA expression compared to the negative control. However, for claudin-1, all the conditions seem to activate the expression of this gene, except low doses of SLST H2 EVs, compared to untreated cells. In contrast, expression levels of MMP-2, a matrix metalloproteinase implicated in tissue remodelling^36^, did not show marked differences for any of the EVs treatment with respect to the control. Regarding the skin immune system cell culture, as Figs. 6B and S1B shows, only SLST A1 EVs triggered a significant expression of the pro-inflammatory cytokine IL-8 compared to the control, while only SLST H1 EVs and high doses of SLST H2 EVs (50 μg/mL) had substantial anti-inflammatory cytokine IL-10 expression. However, for the pro-inflammatory TNFα expression, none of the EVs conditions tested were able to modulate its mRNA levels. As illustrated in Figs. 6C and S1C, SLST A1 EVs are constantly triggering greater activation of the inflammatory cytokines IL-8 and IL-6. Otherwise, for the sebum regulator gene PLIN-2 none of the EVs tested were able to substantially stimulate the up-regulation of this gene expression compared to the untreated control. Additionally, we investigated in parallel the potential of calmodulin 1 (CALM-1) and transient receptor potential channels TRPV-1 and TRPV-4 as biomarkers linked to tissue repair (Fig. S2). However, not statistically significant results were observed.

### EVs derived from *C. acnes* SLST A1 phylotype induce a higher secretion of immunomodulatory mediators in three in vitro skin cell types compared to EVs from *C. acnes* H1 and H2 phylotypes

To confirm the immunomodulatory effects assayed by RT-qPCR in HaCaT, Jurkat and SZ95 in vitro cell types, after 24 h of incubation with different concentrations of SLST A1, H1 and H2 EVs, the protein secretion levels of some cytokines released in the SN were evaluated by Human High Sensitivity Plex Discovery Assay (Eve Technologies).

**Figure 7:**
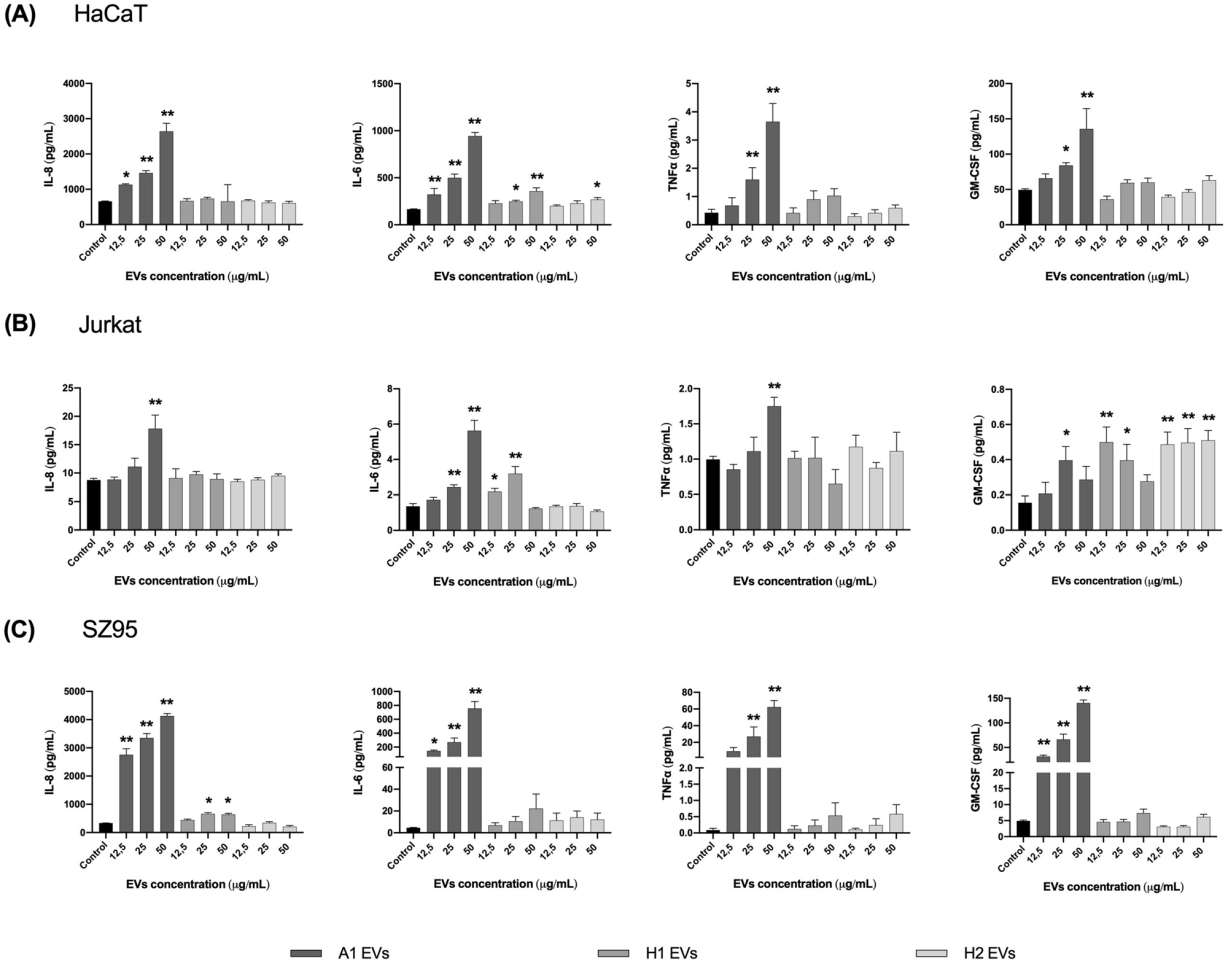
Total protein concentration secreted into the SN after direct incubation with *C. acnes* EVs. Human cells were treated for 24 h with different concentrations (12.5, 25 and 50 μg/mL of *C. acnes* SLST A1, H1 and H2 EVs. Filtered SN was evaluated by Multiplex analysis to measure protein secreted in it. Ranges for the standard curve for each of the biomarkers were: GM-CSF: 0.31–4978.62 pg/mL, TNFα: 0.11–1699.58 pg/mL and IL-6: 0.05–768.83 pg/mL. (**A**) HaCaT (**B**) SZ95 and (**C**) Jurkat immortalized cell lines were used. All data are presented as mean ± standard deviation (SD) of triplicate measurements (*p ≤ 0.05, **p ≤ 0.001 vs. non-stimulated controls).

As shown in Fig. 7A in the HaCaT in vitro culture and following the same pattern as for IL-8 expression level, SLST A1 EVs stimulated the highest secretion of IL-8, IL-6, TNFα and GM-CSF compared to the rest of the EVs, especially when high concentrations (50 μg/mL) of EVs were applied to the keratinocytes. Only 25 and 50 μg/mL for SLST H1 EVs and 50 μg/mL for SLST H2 EVs were able to induce a significant difference in IL-6 secretion compared to the negative control. In the immune system culture, composed of Jurkat cells (Fig. 7B), high doses of SLST A1 EVs were observed to trigger increased secretion of IL-8, IL-6 and TNFα compared to the other conditions, but in the case of GM-CSF, SLST H1 and H2 EVs were the ones involved in the activation of this factor. Regarding the SZ95 cell type (Fig. 7C), IL-8 stimulation correlates with the gene expression data obtained previously. Overall, it can be observed that SLST A1 EVs consistently trigger significant activation of the inflammation-related factors IL-8, IL-6, TNFα and GM-CSF, compared to the negative control. In the case of SLST H1 EVs, only 25 and 50 μg/mL were able to induce significant IL-8 release compared to control.

### Cutibacterium acnes SLST H1 EVs showed to have a better skin sebum inhibition mechanism compared to SLST A1 and H2 EVs

**Figure 8:**
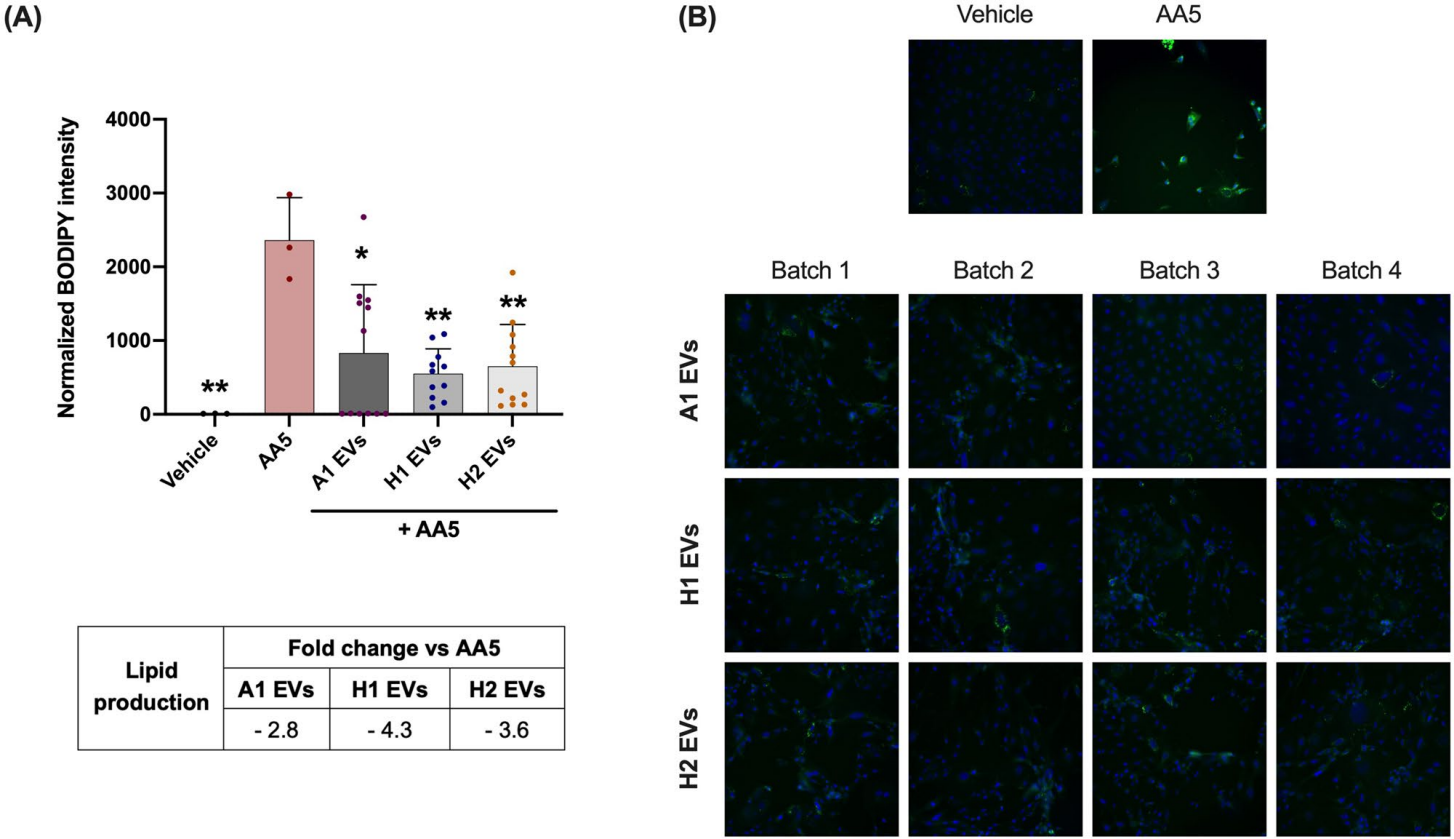
Reduction of lipid production was assessed after treating PCi-SEB with AA5 for 24 h. PCi-SEB were treated during 48 h with AA at 5 µM (AA5) to induce lipid production. Afterwards PCi-SEB were treated for 24 h with 12.5 μg/mL of *C. acnes* SLST A1, H1 and H2 EVs (**A**) Metadata analysis on inter-EVs batches and (**B**) Fluorescence microscopy image of PCi-SEB stained with BODIPY 493/503; negative control (Vehicle), positive control (AA5). All data are presented as mean ± standard deviation (SD) of triplicate measurements (*p ≤ 0.05, **p ≤ 0.001 vs. non-stimulated controls).

The acne-mimicking in vitro model consisted of a 48-h treatment with AA5 as a sebum inducer followed by a 24-h treatment with 12.5 μg/mL of *C. acnes* SLST A1, H1 and H2 EVs. After AA5 incubation, a strong and significant increase in lipid level by about 263-fold was observed, compared to the negative control condition. When PCi-SEB were treated for 24 h with 12.5 μg/mL of *C. acnes* SLST A1, H1 and H2 EVs, they showed a 2.8, 4.3 and 3.6-fold reduction *versus* the AA5 condition, respectively. However, although results showed that the three types of EVs had a significant inhibitory effect on sebum production compared to the positive control, the best sebum inhibitory effect was obtained after SLST H1 EVs treatment, with about fourfold inhibition compared to AA5 condition (Fig. 8).

## Discussion

The Gram-positive bacterium *C. acnes* is one of most abundant skin microbes, playing a pivotal role in maintaining a healthy skin barrier. It has been proposed that *C. acnes* establishes communication with host cells through soluble mediators and EVs, which can permeate through the various layers of the skin, including the epidermis, dermis, and hypodermis. Specifically, upon interaction and internalization into host cells, EVs act as nano-carriers of proteins, metabolites, and nucleic acids. Due to the specific cargo carried by *C. acnes* EVs derived from different phylotypes of *C. acnes*, it is suggested that they may have distinct functions and be responsible for different effects on skin alterations. Currently, *C. acnes* is classified into eight phylotypes (IA1, IA2, IB1, IB2, IB3, IC, II, and III)^37^. Among these, IA1 and IA2 phylotypes are predominantly found in acne lesions, while the phylotype IB is associated with healthy skin without any association to *acne vulgaris*. Affecting approximately 9.4% of the global population, *acne vulgaris* is the 8th most prevalent skin condition. While several treatment options are available, there is still a need for therapeutics against severe cases without posing many side effects^38^. The project aims to investigate the anti-inflammatory and sebum inhibitory properties of EVs secreted by different *C. acnes* phylotypes, specifically SLST A1 (IA1), H1 (IB) and H2 (IB), to assess the potential EVs from certain strains as a safe and promising biotherapy for cutaneous disorders such as *acne vulgaris*.

Research on EVs continues to be a major challenge owing to their intrinsically complex biogenesis, substantial heterogeneity in size and the inherent variability encountered between batches during their production^39–41^.

Moreover, the process to isolate EVs, is tedious and time-consuming and even though novel and faster technologies have emerged^42^, some of them have a toxic nature on in vitro cultures. However, the unique nature of these structures (small, non-synthetic, and non-replicative) enables them to overcome significant hurdles present in the current dermatology and cosmetic field. These barriers include skin penetration, potential side effects from chemicals, and the maintenance of symbiosis and homeostasis with the skin microbiota.

In the following work, Brucella broth was employed for bacterial culture and EVs were isolated following the traditional filtration and ultracentrifugation method. Morphological evaluations conducted using TEM and NTA technology affirmed the presence of closed-spherical structures within the diameter range of 96.8–135.3 nm, consistent with findings from Chudzik et al.^22^. These imaging results indicated no significant differences among the various SLST phylotypes.

To confirm the specific localization of EVs after 24 h of treatment, we opted to test SLST A1 EVs in both HaCaT and SZ95 cell lines. This choice was influenced by several factors: the similarity in membrane composition among EVs, previously observed internalization of *C. acnes* EVs in keratinocytes, their ability to easily diffuse through the sebum layer and establish direct contact with sebocytes, and the practicality of labeling SLST A1 EVs with rhodamine B-R18, since SLST A1 strain has the highest EV production yield.

However, the effectiveness, potential harm, or limited functionality of EVs in the context of *acne vulgaris* pathology hinges on the specific cargo they transport, a cargo directly influenced by their bacterial origin. Therefore, we conducted a proteomic analysis of *C. acnes* EVs to explore the connection between their protein content and the skin microbiota. In agreement with Chudzik et al.^22^, a Venn diagram showed that SLST A1 EVs contain the highest number of proteins, almost four times more proteins than in SLST H1 EVs and nine times more than in SLST H2 EVs. Even though the same amount of EVs (8 μg) was consistently analyzed across replicates, the raw data reveals variations in the number of proteins identified. As reported in prior publications^43,44^, these variations could arise from differences in EV production yields and EV size. Therefore, apart from sharing a conserved core EV proteome, it is suggested that factors beyond the mere presence of a protein in the overall proteome influence the determination of strain-specific EV protein content. In this specific scenario, we also hypothesize that the increased number of proteins in SLST A1 EVs may be attributed to the higher prevalence of pathogenic proteins carried by EVs of SLST A1 phylotype, as opposed to SLST H1 and H2 EVs. In fact, McDowell A. et al., 2013 found that certain strains of *C. acnes* (specifically type IA1) could secrete several virulence factors that enhanced the inflammatory response. Although genetic studies of the *C. acnes* strains used in this work are lacking, previous research has indicated that SLST H1 exhibits beneficial properties, implying a protective effect against *acne vulgaris* development^3^. This fact could be related to a different gene regulation, post-translational modulation, or the loss of certain virulence factors. Based on this last assumption, the mass spectrometry analysis revealed the presence of 25 virulence factors (related to adhesion, biofilm formation, etc.) in SLST A1 EVs, while only 2 were identified in SLST H1 EVs and none in SLST H2 EVs.

The proteomic analysis revealed the presence of several proteins common to all types of *C. acnes* EVs, which are involved in facilitating the transfer of multiple proteins engaged in various biochemical processes and metabolism between cells. This finding confirms the carrier function of EVs and validates their role in cellular transport. Moreover, EVs are used as a way of exchanging cell surface substances and improving bacterial survival during infections^45^ which made it possible to identify several proteins used in antibiotic resistance suggesting that *C.* acnes EVs are also involved in bacterial survival against external threats. In addition, considering the oily sebum niche and characteristics of *C. acnes*, the EVs of all tested strains were found to contain common proteins involved in lipolysis, such as lysophospholipase and triacylglycerol lipase. Although these enzymes are typically regarded as potential virulence factors, lipases play a crucial role in *C. acnes* for maintaining healthy skin. They metabolize sebum and release fatty acids, which are necessary to preserve the skin’s natural acidity, serving as a natural barrier against harmful pathogens and contributing to innate skin immunity^46^.

Taking into consideration the analysis of *C. acnes* phylotype-exclusive proteins for SLST A1, H1 and H2 EVs, significant differences were found in accordance with previous studies, pointing to *C. acnes* phylogroup IA1 as an acne-prone group and *C. acnes* phylogroup IB as a protective commensal group^3,8,11,13,21,22,37,47–49^. As for SLST A1 EVs, its protein profile revealed a great number of proteins involved in bacterial competition and antibiotic resistance (hydrolase, Ppx/GppA phosphatase family, Metallo-ß-lactamase domain protein and histidine kinase)^8^, biofilm formation (glycosyltransferase and serine/threonine-protein kinase) and virulence (porphyrin, sialidase, HtaA domain protein, phosphoesterase, hydrolase and sigma factor SigA) which were not detected in SLST H1 or H2 EVs. Regarding the exclusive proteins of SLST H1 EVs, different proteins directly involved in fatty acid catabolism were found, such as acyl-CoA dehydrogenase^50^ and a putative two-component sensor kinase which upregulates the hydrolysis of sebum triglycerides secreting free fatty acids. Indirectly, the N-acetyl-gamma-glutamyl-phosphate reductase, a protein involved in the L-Arginine biosynthetic pathway, was also found as part of SLST H1 EVs. In this regard, L-arginine assists in the protection of the skin against free radicals, increases the skin's visible hydration levels, and potentially supports collagen production^51^. These findings collectively suggest that EVs share functionalities with their originating bacteria, highlighting EVs as a mechanism through which different *C. acnes* strains exert their effects. This aligns with previous studies^21,52^ which have also emphasized the role of EVs in mediating the impact of *C. acnes* strains.

On the other hand, the differences observed at the protein level among the three SLST *C. acnes* EVs studied are consistent with the findings of Chudzik et al.^22^. They also reported differences at the lipidomic level when comparing similar SLST *C. acnes* EVs phylotypes.

The next step in our study was to investigate if the presence of unique peptides in the different types of *C. acnes* EVs would correspond, as anticipated, to different functionalities within the host. For this purpose, we used a simplified in vitro system that represents the various cell types involved in the pathophysiology of *acne vulgaris*. During *acne vulgaris* development the effects are not limited to the external skin layer; sebocytes and immune cells also contribute significantly to this process^53^. Unlike many publications that primarily focus on keratinocytes, this study evaluates additional cell types, such as sebocytes and immune cells. By incorporating multiple cell types, the study intends to represent all key players in skin research moving closer to representing the complex dynamics occurring in real skin. In order to achieve that, various in vitro assays were conducted. Cytotoxicity assays showed that doses of *C. acnes* EVs up to 50 μg/mL did not significantly affect cell viability of HaCaT and SZ95 cell types, indicating their safety for testing in in vitro skin cultures. Comparing both cell types, it was also observed that sebocytes exhibited a higher level of responsiveness compared to the keratinocytes. This can be attributed to the sebocytes' protected location within the sebaceous gland in the dermis, while keratinocytes are exposed and have continuous interactions with skin microorganisms.

The different *C. acnes* EVs were directly incubated for 24 h in the different in vitro skin cultures: a) HaCaT cells to mimic the epidermis composed mainly of keratinocytes; b) the SZ95 cell culture to mimic the sebaceous gland environment and c) Jurkat cells selected to gain insights into the disruption of the skin barrier, which is a key characteristic of inflammatory skin diseases. The expression levels of different regulatory genes were measured by RT-qPCR and the results revealed that, in line with findings by McDowell et al.^54^, SLST A1 EVs consistently induce higher activation of pro-inflammatory cytokines in all in vitro skin cultures. This could be due to the presence of specific virulence factors found in the proteomic analysis for SLST A1 EVs. On the other side, in the Jurkat cell type, SLST H1 EVs and 50 μg/mL dose of SLST H2 EVs induced a significant expression of the anti-inflammatory cytokine IL-10, reinforcing the possible protective role of these two non-pathogenic strains during *acne vulgaris* development.

To cross-check the immunomodulatory effects assayed by gene expression, the protein secretion levels of certain cytokines released in the SN of the in vitro cultures were evaluated by Multiplex. The results confirmed that *C. acnes* SLST A1 EVs induce a greater secretion of immunomodulatory mediators in all three in vitro skin cultures compared to those induced by *C. acnes* SLST H1 and H2 EVs. According to the latter results, it was determined that SLST A1 EVs consistently triggered a higher activation of the inflammation-related factors IL-8, IL-6, TNFα and GM-CSF, compared to SLST H1 and H2 EVs.

The pathophysiology of *acne vulgaris* involves not only local inflammation but also an excessive production of sebum by sebocytes. This overproduction of sebum leads to the proliferation of specific strains of *C. acnes*, causing a disruption in the skin's natural balance (known as dysbiosis) and ultimately triggering the development of *acne vulgaris*^3,55^*.* In fact, one of the most prescribed treatments for dermatologists against *acne vulgaris* is isotretinoin, a retinoid which causes the apoptosis of sebocytes and reduces therefore the amount of sebum^56^. However, the regulation of sebum and its connection to the development of *acne vulgaris* through the influence of the skin microbiota is a relatively new area of research, and there is a limited amount of literature available on this topic. Therefore, an acne-mimicking skin model with PCi-SEB and AA5 as sebum inductor, was used to explore the effect of *C. acnes* EVs on sebum regulation. After 24-h treatment with 12.5 μg/mL of *C. acnes* SLST A1, H1 and H2 EVs, results showed that the three types of EVs had a significant inhibitory effect on sebum production compared to the positive control, highlighting that the best inhibition was obtained after SLST H1 EVs treatment, with inhibition by about 4-folds compared to the positive control. These findings were anticipated and align with the proteomic analysis, which characterized SLST H1 EVs as carrying exclusive proteins with lipidomic effects.

To our knowledge, this study represents a significant advancement in the understanding of *C. acnes* EVs and its potential implications in *acne vulgaris* treatment. The study includes proteomic characterization of three different phylotypes of *C. acnes* EVs and investigates their effects in various in vitro skin cultures. The findings of this research suggest that *C. acnes* EVs exhibit more complex functionalities beyond being simple lipid spheres. Notably, SLST H1 EVs demonstrate promising bioactive properties as nanocarriers, exhibiting the ability to mitigate *acne vulgaris* symptoms through dual mechanisms: reducing inflammation and modulating sebum production. Further research is required to fully comprehend the therapeutic potential of *C. acnes* EVs and their application in *acne vulgaris* treatment.

## Conclusion

The findings of this project suggest that *C. acnes* EVs play a role in the communication between the skin microbiota and the host, particularly in the context of skin conditions like *acne vulgaris*. While further scientific investigation is necessary to substantiate these findings, the results highlight the protective nature of SLST H1 EVs and their potential as a natural treatment option to alleviate symptoms associated with inflammation and oily skin. To gain a deeper understanding and establish a comprehensive framework, additional experiments involving primary cell lines, in vivo animal models, stability testing of EVs in gel creams, and clinical trials are imperative. Such endeavors will provide valuable insights into the functional mechanisms of *C. acnes* EVs on the skin and their potential applications as treatment modalities.

### Supplementary Information

Supplementary Information.

## Data Availability

The raw data from proteomic analysis are included as Supporting Tables S1, S2 and S3. The original R scripts for plotting the extracellular vesicle data are available on Bitbucket. (https://bitbucket.org/synbiolab/fabrega_vesicles/src/master/).

## Abbreviations

*C*. *acnes*: *Cutibacterium* *acnes*
EVs: Extracellular vesicles
PBS: Phosphate-buffered saline
SN: Supernatant
TEM: Transmission electron microscopy
NTA: Nanoparticle tracking analysis
SDS-PAGE: Sodium dodecyl sulfate-polyacrylamide gel electrophoresis
PBS: Phosphate-buffered saline
CPS: Cycles per second
DDA: Data-dependent acquisition
AGC: Auto gain control
HCD: High-energy collision dissociation
FDR: False discovery rate
XIC: Extracted ion current
ATCC: American Type Culture Collection
DMEM: Dulbecco’s Modified Eagle Medium
FBS: Fetal bovine serum
EGFr: Recombinant human epidermal growth factor
CaCl_2_: Calcium chloride
RPMI: Roswell Park Memorial Institute medium
RT: Room temperature
WGA: Wheat germ agglutinin
RT-qPCR: Quantitative real time-PCR analysis
PCi-SEB: Human iPSC-derived sebocytes
AA5: 5 μM of arachidonic acid
SD: Standard deviation
GO: Gene Ontology
IL: Interleukin
CITE: Biofilm matrix dynamics
