# Author Correction: New insights into the role of *Cutibacterium acnes*-derived extracellular vesicles in inflammatory skin disorders

**DOI:** 10.1038/s41598-023-45727-7

**Published:** 2023-11-08

**Authors:** Maria Pol Cros, Júlia Mir-Pedrol, Lorena Toloza, Nastassia Knödlseder, Julien Maruotti, Christos C. Zouboulis, Marc Güell, Maria-José Fábrega

**Affiliations:** 1 Department of Medicine and Life Sciences, Universitat Pompeu Fabra, Barcelona, Spain; 2 Fachbibliothek Mathematik und Physik, Universitaet Tuebingen, Tubingen, Baden-Württemberg Germany; 3 Phenocell, Grasse, France; 4 Hochschulklinik für Dermatologie, Venerologie und Allergologie, Immunologisches Zentrum, Städtisches Klinikum Dessau, Medizinische Hochschule Brandenburg Theodor Fontane und Fakaltät für Gesundheitswissenschaften Brandenburg, Auenweg, Germany

Correction to: *Scientific Reports* 10.1038/s41598-023-43354-w, published online 25 September 2023

The Funding section in the original version of this Article was incomplete.

“This work was funded and supported by the University Pompeu Fabra-Excelencia Maria de Maeztu Post-doctoral program (CEX 2018-0007392-M). NK is funded by a Maria Maetzu-UPF fellowship (Catalan Government) and by the Sociedad Española de Químicos Cosméticos (SEQC) and MJF is funded by a Juan de la Cierva Fellowship from the Spanish Government (Award FJC 2018-037096-I).”

now reads:

“This work was funded and supported by the University Pompeu Fabra-Excelencia Maria de Maeztu Post-doctoral program (CEX 2018-0007392-M). NK is funded by a Maria Maetzu-UPF fellowship (Catalan Government) and by the Sociedad Española de Químicos Cosméticos (SEQC) and MJF is funded by a Juan de la Cierva Fellowship from the Spanish Government (Award FJC 2018-037096-I). The proteomics analyses were performed in the CRG/UPF Proteomics Unit which is part of the Proteored, PRB3 and is supported by grant PT17/0019, of the PE I+D+i 2013-2016, funded by ISCIII and ERDF.”

The original Article has been corrected.

