# Author Correction: New insights into the role of *Cutibacterium acnes*-derived extracellular vesicles in inflammatory skin disorders

**DOI:** 10.1038/s41598-024-53555-6

**Published:** 2024-02-06

**Authors:** Maria Pol Cros, Júlia Mir-Pedrol, Lorena Toloza, Nastassia Knödlseder, Julien Maruotti, Christos C. Zouboulis, Marc Güell, Maria-José Fábrega

**Affiliations:** 1 Department of Medicine and Life Sciences, Universitat Pompeu Fabra, Barcelona, Spain; 2 Quantitative Biology Center, University of Tuebingen, Tuebingen, Baden-Württemberg Germany; 3 Phenocell, Grasse, France; 4 Hochschulklinik für Dermatologie, Venerologie und Allergologie, Immunologisches Zentrum, Städtisches Klinikum Dessau, Medizinische Hochschule Brandenburg Theodor Fontane und Fakaltät für Gesundheitswissenschaften Brandenburg, Auenweg, Germany

Correction to: *Scientific Reports* 10.1038/s41598-023-43354-w, published online 25 September 2023

The original version of this Article contained an error in Affiliation 2, which was incorrectly given as ‘Fachbibliothek Mathematik und Physik, Universitaet Tuebingen, Tubingen, Baden-Württemberg, Germany’. The correct affiliation is listed below.

Quantitative Biology Center, University of Tuebingen, Tuebingen, Baden-Württemberg, Germany

The original Article has been corrected.

