# Supplementary material for: New insights into the role of Cutibacterium acnes-derived extracellular vesicles in inflammatory skin disorders
Source: Sci Rep. 2023 Sep 25;13:16058. doi: 10.1038/s41598-023-43354-w (PMC10520063; doi:10.1038/s41598-023-43354-w)
Supplement: Supplementary file 1 — Supplementary Information. [file 41598_2023_43354_MOESM1_ESM.pdf]

SUPPORTING INFORMATION

Table S1. List of proteins identified exclusively in *C. acnes*-derived A1 EVs under anaerobic conditions

Gene name	Accession number	Protein name	GO - Biological process	GO - Molecular function	GO - Cellular component
pyrK PPA1001 cbac_05440	WP_002517886.1	Dihydroorotate dehydrogenase B	Biosynthetic process	Binding	Membrane
cbac_03745	WP_073842171.1	PTS sugar transporter	Transport	Transferase activity	Membrane
PPA1099 cbac_05960	WP_002514993.1	PAC2 family protein	Protein processing	Unknown	Cytoplasm
pyrD cbac_05445	WP_002517834.1	Dihydroorotate dehydrogenase	Biosynthetic process	Dehydrogenase activity	Cytoplasm
PPA2168 cbac_11750	WP_002516248.1	Formimidoylglutamate deiminase	Metabolic process	Peptidase activity	Cytoplasm
cbac_02600	WP_002531152.1	Copper homeostasis protein CutC	Cellular homeostasis	Binding	Unknown
folP PPA0271 cbac_01520	WP_002517047.1	Dihydropteroate synthase	Biosynthetic process	Binding	Cytoplasm
PPA0313 cbac_01745	WP_002517195.1	NAD-dependent malic enzyme	Metabolic process	Dehydrogenase activity	Cytoplasm
hisF HMPREF0675_4424	WP_002516482.1	Imidazole glycerol phosphate synthase subunit HisF	Biosynthetic process	Lyase activity	Cytoplasm
PPA0464 cbac_02555	WP_002530084.1	Sugar phosphate isomerase/epimerase	Biosynthetic process	Isomerase activity	Membrane
PPA0464 cbac_02555	WP_002530084.1	Sugar phosphate isomerase/epimerase	Biosynthetic process	Isomerase activity	Cytoplasm
PPA1005 cbac_05460	WP_002515547.1	Fructosamine kinase	Protein processing	Kinase activity	Cytoplasm
cbac_02675	WP_002518785.1	Phosphoserine transaminase	Biosynthetic process	Transferase activity	Cytoplasm
ddl cbac_12720	WP_002515704.1	D-alanine--D-alanine ligase	Cell wall organization	Binding	Cytoplasm
thiE cbac_04840	WP_002531442.1	Thiamine-phosphate synthase	Biosynthetic process	Binding	Cytoplasm
PPA0303 cbac_01695	WP_002531197.1	Uroporphyrinogen-III synthase	Biosynthetic process	Synthase activity	Cytoplasm
HMPREF0675_4140	WP_002515011.1	Histidine triad domain protein	Transport	Catalytic activity	Membrane
cobM PPA0421 cbac_02310	WP_002515099.1	Precorrin-4 C	Biosynthetic process	Transferase activity	Unknown
hutI HMPREF0675_5232	WP_002516336.1	Imidazolonepropionate	Metabolic process	Binding	Cytoplasm
PPA1225 cbac_06630	WP_002524933.1	Pyridoxal kinase	Biosynthetic process	Kinase activity	Cytoplasm
PPA1015 cbac_05515	WP_002515534.1	Aldose epimerase	Metabolic process	Binding	Membrane
PPA1982 cbac_10760	WP_002530665.1	Phosphatase PAP2 family protein	Lipid metabolism	Phosphatase activity	Membrane
PPA0525 cbac_02915	WP_002516665.1	DNase	Degradation	Hydrolase activity	Cytoplasm
PPA1009 cbac_05480	WP_002513709.1	Possible transcriptional regulator	Transcription	Molecular function regulator	Cytoplasm
PPA1629 cbac_08845	WP_002517032.1	6-phosphogluconate dehydrogenase, decarboxylating	Metabolic process	Dehydrogenase activity	Cytoplasm
PPA2286 cbac_12435	WP_002515771.1	Phosphoglucomutase	Metabolic process	Transferase activity	Cytoplasm
PPA0106 cbac_00580	WP_002512819.1	ABC transporter substrate-binding protein	Transport	Binding	Membrane
tsaB PPA1784 cbac_09710	WP_002531267.1	Peptidase, family M22	Translation	Transferase activity	Membrane
tsaB PPA1784 cbac_09710	WP_002531267.1	Peptidase, family M22	Translation	Transferase activity	Cytoplasm
idi cbac_11445	WP_002530756.1	Isopentenyl-diphosphate Delta-isomerase	Biosynthetic process	Hydrolase activity	Cytoplasm
PPA2156 cbac_11695	WP_002530776.1	Dipeptide ABC transporter ATP-binding protein	Transport	Transporter activity	Membrane
murQ HMPREF9578_00165	WP_002513573.1	N-acetylmuramic acid 6-phosphate etherase	Metabolic process	Lyase activity	Cytoplasm
PPA2214 cbac_12030	WP_002530902.1	Electron transfer flavoprotein subunit alpha	Metabolic process	Transferase activity	Unknown
HMPREF9206_1221	WP_002512973.1	Phosphoglycerate mutase family protein	Metabolic process	Catalytic activity	Cytoplasm

menC PPA0902 cbac_04915	WP_002515608.1	o-succinylbenzoate synthase	Biosynthetic process	Synthase activity	Cytoplasm
cmk HMPREF0675_4273	WP_002516760.1	Cytidylate kinase	Metabolic process	Kinase activity	Cytoplasm
metK HMPREF0675_4255	WP_002517758.1	S-adenosylmethionine synthase	Metabolic process	Binding	Cytoplasm
cbac_03780	WP_002530937.1	Exo-alpha-sialidase	Lipid metabolism	Sialidase activity	Cytoplasm
PPA1781 cbac_09695	WP_002514786.1	8-oxo-dGTP diphosphatase	DNA repair	Hydrolase activity	Cytoplasm
PPA1544 cbac_08415	WP_002514359.1	Conserved protein	Protein processing	Binding	Cytoplasm
PPA1950 cbac_10560	WP_002523392.1	Cell division protein DedD	Metabolic process	Deaminase activity	Membrane
coaD HMPREF9578_02029	WP_002514272.1	Phosphopantetheine adenylyltransferase	Biosynthetic process	Transferase activity	Cytoplasm
PPA2116 cbac_11450	WP_002530757.1	Serine protease	Biosynthetic process	Peptidase activity	Membrane
PPA0443 cbac_02415	WP_002516675.1	Cobalamin-binding protein	Transport	Binding	Cytoplasm
HMPREF0675_3579	WP_002517279.1	Transcriptional regulator, TetR family	Transcription	Molecular function regulator	Nucleoid
HMPREF0675_5283	WP_002516338.1	FAD dependent oxidoreductase	Metabolic process	Oxidoreductase activity	Cytoplasm
PPA1366 cbac_07465	WP_002516499.1	FAA hydrolase family protein	Metabolic process	Catalytic activity	Cytoplasm
manA PPA0008 cbac_00045	WP_002515893.1	Mannose-6-phosphate isomerase	Metabolic process	Isomerase activity	Cytoplasm
cbac_06585	WP_002516778.1	HAD family hydrolase	Protein processing	Hydrolase activity	Cytoplasm
PPA1377 cbac_07530	WP_002531311.1	RNA helicase	Unknown	Binding	Cytoplasm
PPA0603 cbac_03350	WP_007400924.1	Putative transcriptional regulator	Transcription	Molecular function regulator	Unknown
HMPREF0675_5327	WP_002518141.1	Transcriptional regulator, MarR family	Transcription	Molecular function regulator	Cytoplasm
gyrA PPA0010 cbac_00055	WP_002530866.1	DNA gyrase subunit A	DNA replication	Binding	Cytoplasm
cbac_02560	WP_002524726.1	Sugar phosphate isomerase/epimerase	Metabolic process	Isomerase activity	Cytoplasm
cbac_04330	WP_073885323.1	HtaA domain protein	Translation	Binding	Membrane
PPA1181 cbac_06395	WP_002516773.1	Shikimate 5-dehydrogenase	Metabolic process	Dehydrogenase activity	Cytoplasm
prcB PPA1206 cbac_06530	WP_002516776.1	20S proteasome beta-subunit	Metabolic process	Peptidase activity	Cytoplasm
PPA0947 cbac_05145	WP_002513775.1	DNA-binding response regulator	Transcription	Molecular function regulator	Cytoplasm
HMPREF0675_5322	WP_002516444.1	Putative 3-methyladenine DNA glycosylase	DNA replication	Binding	Nucleoid
HMPREF0675_5322	WP_002516444.1	Putative 3-methyladenine DNA glycosylase	DNA replication	Binding	Cytoplasm
PPA1371 cbac_07495	WP_002516506.1	Putative transferase	Biosynthetic process	Transferase activity	Unknown
PPA2273 cbac_12370	WP_002519568.1	Adenosine deaminase	DNA damage	Deaminase activity	Cytoplasm
gatA cbac_06100	WP_002531385.1	Glutamyl-tRNA	Translation	Hydrolase activity	Cytoplasm
PPA1631 cbac_08855	WP_002517011.1	Thioesterase	Regulation of biological process	Catalytic activity	Cytoplasm
cbac_10785	WP_002530669.1	ATPase	Energy process	Hydrolase activity	Membrane
PPA1290 cbac_07010	WP_002531340.1	2,3-diaminopropionate biosynthesis protein SbnA	Biosynthetic process	Synthase activity	Cytoplasm
fmt HMPREF0675_4257	WP_002517988.1	Methionyl-tRNA formyltransferase	Translation	Transferase activity	Cytoplasm
tmk HMPREF0675_3289	WP_002517149.1	Thymidylate kinase	Biosynthetic process	Kinase activity	Cytoplasm
ung PPA0558 cbac_03120	WP_002519227.1	Uracil-DNA glycosylase	DNA repair	Glycosylase activity	Cytoplasm
PPA1277 cbac_06910	WP_002531349.1	ABC transporter substrate-binding protein	Transport	Binding	Membrane
cbac_09495	WP_041444224.1	Phosphoesterase	Pathogenesis (Virulence)	Hydrolase activity	Cytoplasm
lysA PPA1259 cbac_06800	WP_002516817.1	Diaminopimelate decarboxylase	Biosynthetic process	Decarboxylase activity	Cytoplasm
rpoZ HMPREF1162_0201	WP_002516821.1	DNA-directed RNA polymerase subunit omega	Transcription	Polymerase activity	Nucleoid
HMPREF9578_00185	WP_002516610.1	ACT domain-containing protein	Metabolic process	Structural molecule activity	Cytoplasm
PPA0563 cbac_03150	WP_011183699.1	Conserved protein containing thioredoxin domain	Metabolic process	Reductase activity	Cytoplasm

cbac_01645	WP_002523010.1	PTS glucose transporter subunit IIB	Transport	Kinase activity	Membrane
PPA0548 cbac_03045	WP_002516725.1	Hydrolase, Ppx/GppA phosphatase family	Metabolic process	Hydrolase activity	Cytoplasm
PPA2167 cbac_11745	WP_002516352.1	Allantoate amidohydrolase (AAH, allantoinase)	Metabolic process	Hydrolase activity	Cytoplasm
PPA0814 cbac_04460	WP_002519094.1	Putative gluconeogenesis factor	Cell wall organization and cell shape	Transferase activity	Cytoplasm
map PPA1833 cbac_09965	WP_002523458.1	Methionine aminopeptidase	Protein processing	Peptidase activity	Cytoplasm
mfd cbac_03010	WP_002518816.1	Transcription-repair-coupling factor	Transcription	Binding	Cytoplasm
PPA1711 cbac_09310	WP_002522145.1	Short chain dehydrogenase	Lipid metabolism	Oxidoreductase activity	Cytoplasm
PPA1048 cbac_05685	WP_002515631.1	PglZ domain-containing protein	Cellular homeostasis	Catalytic activity	Cytoplasm
cbac_01755	WP_002518719.1	Sugar phosphate isomerase/epimerase	Metabolic process	Isomerase activity	Cytoplasm
cbac_02605	WP_002518777.1	Tat pathway signal protein	Transport	Transolcase activity	Membrane
cbac_12735	WP_002518540.1	Chromosome partitioning protein ParB	DNA replication	Binding	Cytoplasm
dnaA HMPREF0675_3000	WP_002515747.1	Chromosomal replication initiator protein DnaA	DNA replication	Binding	Cytoplasm
rbpA PPA0820 cbac_04490	WP_002513867.1	RNA polymerase-binding protein RbpA	Transcription	Binding	Unknown
gatB HMPREF0675_4186	WP_002516598.1	Aspartyl/glutamyl-tRNA	Translation	Transferase activity	Cytoplasm
pgl PPA1565 cbac_08515	WP_002521397.1	6-phosphogluconolactonase	Metabolic process	Phosphogluconolactonase activity	Unknown
PPA1083 cbac_05885	WP_002523969.1	Glycine cleavage system protein H	Metabolic process	Transferase activity	Membrane
ligA cbac_08830	WP_002531030.1	DNA ligase	DNA repair	Ligase activity	Cytoplasm
ligA cbac_08830	WP_002531030.1	DNA ligase	DNA replication	Ligase activity	Cytoplasm
purK PPA1702 cbac_09260	WP_002531235.1	N5-carboxyaminoimidazole ribonucleotide synthase	Biosynthetic process	Synthase activity	Unknown
PPA1031 cbac_05600	WP_002515632.1	RNA polymerase principal sigma factor HrdD	Transcription	Binding	Cytoplasm
whiA HMPREF0675_3880	WP_002515546.1	Probable cell division protein WhiA	Cell division	Binding	Cytoplasm
cbac_01795	WP_002531193.1	Histidine phosphatase family protein	Regulation of biological process	Isomerase activity	Cytoplasm
cbac_02850	WP_002518803.1	Molybdopterin-guanine dinucleotide biosynthesis protein	Biosynthetic process	Binding	Cytoplasm
trpC PPA1130 cbac_06135	WP_002516614.1	Indole-3-glycerol phosphate synthase	Biosynthetic process	Synthase activity	Cytoplasm
cbac_00405	WP_002515702.1	ABC transporter ATP-binding protein	Transport	Binding	Membrane
PPA1364 cbac_07445	WP_002517908.1	IclR family transcriptional regulator	Transcription	Molecular function regulator	Cytoplasm
PPA0655 cbac_03620	WP_002515176.1	Antitoxin HicB	Response to stimulus	Binding	Unknown
PPA0055 cbac_00300	WP_002515807.1	DNA-binding response regulator	Transcription	Molecular function regulator	Nucleoid
rsmI PPA0524 cbac_02910	WP_002516667.1	Ribosomal RNA small subunit methyltransferase I	Response to stimulus	Transferase activity	Cytoplasm
PPA1824 cbac_09925	WP_009640388.1	Glycosyl transferase	Biosynthetic process	Transferase activity	Cytoplasm
PPA2237 cbac_12170	WP_002516470.1	1-acyl-sn-glycerol-3-phosphate acyltransferase	Biosynthetic process	Transferase activity	Cytoplasm
PPA1146 cbac_06210	WP_011183804.1	Beta-glucosidase	Metabolic process	Hydrolase activity	Cytoplasm
cbac_05620	WP_002515456.1	Alpha/beta hydrolase	Degradation	Hydrolase activity	Membrane
HMPREF0675_5088	WP_002518085.1	Phosphomannomutase	Biosynthetic process	Hydrolase activity	Cytoplasm
cbac_06220	WP_041444150.1	Permease IIC component	Transport	Transferase activity	Membrane
PPA0082 cbac_00450	WP_002515718.1	Hypothetical membrane associated protein	Transport	Transporter activity	Membrane
rnhA PPA1729 cbac_09405	WP_002531248.1	Ribonuclease H	Metabolic process	Binding	Cytoplasm
PPA1898 cbac_10295	WP_002531304.1	Exodeoxyribonuclease III	DNA repair	Ribonuclease activity	Cytoplasm
PPA1069 cbac_05795	WP_002517916.1	Amino acid permease	Transport	Transporter activity	Membrane
PPA2185 cbac_11850	WP_002516309.1	Acyl dehydratase	Metabolic process	Isomerase activity	Cytoplasm

cbac_09895	WP_002525147.1	Glyco_hydro_35 domain-containing protein	Metabolic process	Hydrolase activity	Cytoplasm
cbac_06995	WP_002524911.1	Peptide synthetase	Biosynthetic process	Synthase activity	Cytoplasm
uvrB cbac_04410	WP_002518347.1	UvrABC system protein B	Response to stimulus	Binding	Cytoplasm
PPA1118 cbac_06075	WP_009640307.1	Methionine synthase vitamin-B12 independent	Biosynthetic process	Transferase activity	Cytoplasm
rsmG cbac_12765	WP_002518545.1	Ribosomal RNA small subunit methyltransferase G	Translation	Transferase activity	Cytoplasm
PPA0120 cbac_00670	WP_002530833.1	EXLDI protein	Unknown	Unknown	Unknown
PPA0624 cbac_03465	WP_002530925.1	N-acetyltransferase	Metabolic process	Transferase activity	Cytoplasm
PPA1166 cbac_06315	WP_002517766.1	Bifunctional	Metabolic process	Hydrolase activity	Cytoplasm
PPA2037 cbac_11045	WP_002514480.1	MerR family DNA-binding transcriptional regulator	Transcription	Molecular function regulator	Cytoplasm
PPA1186	WP_002513526.1	PPK2 domain-containing protein	Metabolic process	Kinase activity	Cytoplasm
PPA1616 cbac_08775	WP_002517026.1	DNA helicase	DNA replication	Hydrolase activity	Cytoplasm
PPA0348 cbac_01935	WP_002517076.1	Conserved protein, putative glycine cleavage T-protein	Metabolic process	Unknown	Unknown
PPA1488 cbac_08115	WP_002520231.1	Arabinose operon protein AraM	Biosynthetic process	Oxidoreductase activity	Cytoplasm
PPA1524 cbac_08315	WP_009640297.1	6-phosphogluconolactonase	Metabolic process	Phosphogluconolactonase activity	Cytoplasm
cbac_04925	WP_002515532.1	Isochorismate synthase	Biosynthetic process	Synthase activity	Cytoplasm
cobO PPA0437 cbac_02385	WP_002515069.1	Cob	Biosynthetic process	Transferase activity	Unknown
manA PPA0451 cbac_02480	WP_002517436.1	Mannose-6-phosphate isomerase	Metabolic process	Isomerase activity	Cytoplasm
PPA1085 cbac_05895	WP_002513631.1	MerR family transcriptional regulator	Transcription	Molecular function regulator	Cytoplasm
PPA2254 cbac_12250	WP_002516466.1	Amino acid permease	Transport	Transporter activity	Membrane
PPA0639 cbac_03540	WP_002530928.1	Glycogen synthase	Biosynthetic process	Transferase activity	Cytoplasm
PPA0177 cbac_00965	WP_002530809.1	APC family permease	Transport	Transporter activity	Membrane
cbac_12740	WP_002518541.1	ParA family protein	Cell division	Binding	Cytoplasm
PPA0685 cbac_03785	WP_002515306.1	Sialidase	Metabolic process	Sialidase activity	Cytoplasm
cobI PPA0420 cbac_02305	WP_002531167.1	Precorrin-2 C	Biosynthetic process	Transferase activity	Cytoplasm
PPA1464 cbac_07995	WP_009640171.1	Methylase	Translation	Transferase activity	Cytoplasm
nhaA HMPREF0675_3274	WP_002524662.1	Na	Cellular homeostasis	Transporter activity	Membrane
PPA2031 cbac_11015	WP_002530684.1	ABC transporter associated permease	Transport	Transporter activity	Membrane
mraY HMPREF9578_00542	WP_002513927.1	Phospho-N-acetylmuramoyl-pentapeptide-transferase	Cell wall organization and cell shape	Transferase activity	Membrane
HMPREF9578_00411	WP_002521769.1	Metallo-beta-lactamase domain protein	Regulation of biological process	Hydrolase activity	Cytoplasm
cbac_03830	WP_002523706.1	Cytochrome c oxidase assembly protein	Protein processing	Unknown	Membrane
PPA1365 cbac_07460	WP_011183848.1	CPBP family intramembrane metalloprotease	Protein processing	Peptidase activity	Membrane
PPA0557 cbac_03110	WP_002515113.1	Transporter	Transport	Transporter activity	Membrane
thiL PPA1357 cbac_07420	WP_002515338.1	Thiamine-monophosphate kinase	Biosynthetic process	Binding	Cytoplasm
cbac_11435	WP_002530754.1	Histidine kinase	Regulation of biological process	Kinase activity	Membrane
HMPREF0675_4955	WP_002515985.1	Enoyl-CoA hydratase/isomerase family protein	Unknown	Isomerase activity	Cytoplasm
tyrS cbac_07675	WP_009640057.1	Tyrosine--tRNA ligase	Translation	Ligase activity	Cytoplasm
thrC HMPREF0675_4320	WP_002518907.1	Threonine synthase	Biosynthetic process	Synthase activity	Cytoplasm
PPA1258 cbac_06795	WP_002517921.1	Homoserine dehydrogenase	Biosynthetic process	Dehydrogenase activity	Cytoplasm
ddl PPA1359 cbac_07430	WP_002531315.1	D-alanine--D-alanine ligase	Cell wall organization and cell shape	Ligase activity	Cytoplasm
PPA0109 cbac_00605	WP_011183602.1	Myosin-crossreactive antigen	Lipid metabolism	Binding	Cytoplasm
cbac_09270	WP_002517608.1	Peptidase E	Metabolic process	Peptidase activity	Cytoplasm

PPA2251 cbac_12235	WP_002518145.1	D-glycerate dehydrogenase	Metabolic process	Dehydrogenase activity	Cytoplasm
cbac_10845	WP_002518064.1	Dihydrodipicolinate synthase family protein	Biosynthetic process	Lyase activity	Cytoplasm
PPA1367 cbac_07470	WP_002516498.1	Thymidine phosphorylase	Metabolic process	Phosphorylase activity	Cytoplasm
purQ HMPREF9578_01524	WP_002514428.1	Phosphoribosylformylglycinamidine synthase subunit PurQ	Biosynthetic process	Synthase activity	Cytoplasm
PPA2322 cbac_12635	WP_002517539.1	Conserved L-arabinose operon protein, hydrolase	Transcription	Hydrolase activity	Cytoplasm
PPA1285 cbac_06985	WP_002517793.1	ATP-grasp domain-containing protein	Energy process	Binding	Cytoplasm
PPA2238 cbac_12175	WP_002516449.1	AraC family transcriptional regulator	Transcription	Molecular function regulator	Cytoplasm
PPA2125 cbac_11505	WP_007401162.1	Polyphosphate glucokinase/transcriptional regulator	Transcription	Kinase activity	Cytoplasm
PPA1905 cbac_10330	WP_002531307.1	2-oxoacid:ferredoxin oxidoreductase subunit beta	Metabolic process	Catalytic activity	Cytoplasm
cobF PPA1920 cbac_10410	WP_002524506.1	Precorrin-6A synthase	Biosynthetic process	Synthase activity	Cytoplasm
PPA2093 cbac_11320	WP_002530734.1	Alpha-ketoacid dehydrogenase subunit beta	Response to stimulus	Dehydrogenase activity	Cytoplasm
PPA0806 cbac_04425	WP_002550711.1	Maleylpyruvate isomerase family mycothiol-dependent enzyme	Metabolic process	Isomerase activity	Cytoplasm
murE PPA0753 cbac_04150	WP_002518243.1	UDP-N-acetylmuramoyl-L-alanyl-D-glutamate--2,6-diaminopimelate ligase	Biosynthetic process	Ligase activity	Cytoplasm
PPA0396	WP_002515101.1	L-serine dehydratase	Metabolic process	Binding	Cytoplasm
PPA1067 cbac_05785	WP_002520491.1	Transcriptional regulator	Transcription	Molecular function regulator	Cytoplasm
PPA1719 cbac_09355	WP_002514720.1	Biotin carboxylase	Lipid metabolism	Ligase activity	Cytoplasm
rplK HMPREF0675_4942	WP_002516066.1	50S ribosomal protein L11	Translation	Structural molecule activity	Ribosome
PPA0081 cbac_00440	WP_002517298.1	Alpha-1,4 glucan phosphorylase	Metabolic process	Phosphorylase activity	Cytoplasm
cbac_02400	WP_002517307.1	Cobalt-precorrin-6A reductase	Biosynthetic process	Reductase activity	Cytoplasm
hemE cbac_01710	WP_002531196.1	Uroporphyrinogen decarboxylase	Biosynthetic process	Decarboxylase activity	Cytoplasm
PPA1442 cbac_07865	WP_002514249.1	UPF0109 protein PPA1442	Unknown	Binding	Unknown
cbac_11690	WP_002516303.1	ABC transporter ATP-binding protein	Transport	Transporter activity	Membrane
pyrG HMPREF0675_4439	WP_002517885.1	CTP synthase	Metabolic process	Binding	Cytoplasm
cbac_05930	WP_002551083.1	Threonylcarbamoyl-AMP synthase	Biosynthetic process	Binding	Cytoplasm
PPA0722 cbac_03995	WP_002521859.1	Glucokinase	Metabolic process	Kinase activity	Cytoplasm
rplT HMPREF9578_02089	WP_002514221.1	50S ribosomal protein L20	Translation	Structural molecule activity	Ribosome
typA bipA PPA2003 cbac_10865	WP_002516314.1	50S ribosomal subunit assembly factor BipA	Translation	Binding	Ribosome
PPA0131 cbac_00730	WP_002515744.1	Glycosyl transferase	Biosynthetic process	Transferase activity	Cytoplasm
pdhA cbac_11325	WP_002530735.1	Pyruvate dehydrogenase	Unknown	Oxidoreductase activity	Unknown
cbac_02450	WP_007400895.1	PTS sugar transporter	Transport	Transporter activity	Membrane
carB cbac_05435	WP_002531412.1	Carbamoyl-phosphate synthase large chain	Biosynthetic process	Synthase activity	Cytoplasm
PPA0286 cbac_01600	WP_002531205.1	Conserved protein, putative cell division inhibitor	Cell division	Catalytic activity	Cytoplasm
PPA0147 cbac_00815	WP_002515854.1	Glycosyltransferase family 4 protein	Unknown	Transferase activity	Cytoplasm
cbac_06440	WP_002524945.1	30S ribosomal protein S13	Translation	Structural molecule activity	Ribosome
HMPREF0675_5340	WP_002516467.1	ErfK/YbiS/YcfS/YnhG	Biosynthetic process	Transferase activity	Membrane
PPA1056 cbac_05725	WP_011183789.1	Conserved protein	Unknown	Binding	Unknown
sigA cbac_05605	WP_002519002.1	RNA polymerase sigma factor SigA	Transcription	Binding	Cytoplasm
ettA PPA1636 cbac_08880	WP_002516980.1	Energy-dependent translational throttle protein EttA	Translation	Transferase activity	Cytoplasm
rbsK PPA0018 cbac_00095	WP_002515739.1	Ribokinase	Metabolic process	Kinase activity	Cytoplasm
cbac_03165	WP_002519222.1	PhoH family protein	Unknown	Binding	Unknown
thiM PPA0885	WP_002531443.1	Hydroxyethylthiazole kinase	Biosynthetic process	Binding	Cytoplasm

cbac_03675	WP_002519174.1	Glutamine synthetase	Biosynthetic process	Ligase activity	Cytoplasm
polA cbac_04270	WP_002523754.1	DNA polymerase I	DNA repair	Binding	Nucleoid
polA cbac_04270	WP_002523754.1	DNA polymerase I	DNA replication	Binding	Nucleoid
PPA0945 cbac_05135	WP_002526277.1	Putative histidine kinase	Regulation of biological process	Kinase activity	Membrane
cbac_12525	WP_002519762.1	Serine/threonine protein kinase	Regulation of biological process	Kinase activity	Cytoplasm
PPA0281 cbac_01575	WP_002517113.1	Conserved membrane spanning protein	Transport	Transporter activity	Membrane
radA PPA0312 cbac_01740	WP_002517527.1	DNA repair protein RadA	DNA repair	Hydrolase activity	Cytoplasm
PPA0342 cbac_01905	WP_002517143.1	Conserved protein	Unknown	Hydrolase activity	Cytoplasm
PPA1639 cbac_08895	WP_002524831.1	ABC transporter	Transport	Transporter activity	Membrane
PPA2096 cbac_11340	WP_009639878.1	Molybdenum cofactor biosynthesis enzyme/coproporphyrinogen III oxidase	Biosynthetic process	Catalytic activity	Cytoplasm
cbac_04120	WP_002518266.1	DUF58 domain-containing protein	Unknown	Structural molecule activity	Membrane
PPA0710 cbac_03930	WP_002530941.1	Cytochrome bc1 complex cytochrome b subunit	Unknown	Reductase activity	Membrane
hrcA HMPREF9578_00410	WP_002513807.1	Heat-inducible transcription repressor HrcA	Response to stimulus	Binding	Cytoplasm
PPA0072 cbac_00395	WP_002525540.1	Membrane associated protein	Transport	Transporter activity	Membrane
PPA2298 cbac_12500	WP_002515857.1	Conserved protein, putative mechanosensitive ion channel	Transport	Transporter activity	Membrane
PPA0732 cbac_04045	WP_002513952.1	Geranylgeranyl pyrophosphate synthase	Biosynthetic process	Transferase activity	Cytoplasm
PPA1671 cbac_09095	WP_002522165.1	Dihydrofolate reductase	Metabolic process	Reductase activity	Cytoplasm
PPA1917 cbac_10395	WP_002515372.1	Hypothetical membrane protein	Transport	Transporter activity	Membrane
PPA0434	WP_002517347.1	ABC transporter ATP-binding protein	Transport	Transferase activity	Membrane
PPA1476 cbac_08060	WP_002516879.1	Glycine betaine transport system permease protein	Transport	Transporter activity	Membrane
PPA1955 cbac_10590	WP_002524492.1	Right-handed parallel beta-helix repeat-containing protein	Unknown	Catalytic activity	Unknown
cbac_04030	WP_002521854.1	Non-specific serine/threonine protein kinase	Regulation of biological process	Kinase activity	Cytoplasm
nuoH HMPREF9578_02182	WP_002514928.1	NADH-quinone oxidoreductase subunit H	Unknown	Oxidoreductase activity	Membrane
cbac_00710	WP_002526015.1	DUF2029 domain-containing protein	Unknown	Structural molecule activity	Membrane
PPA0128 cbac_00715	WP_011183607.1	Membrane spanning protein	Biosynthetic process	Transferase activity	Membrane
PPA2033 cbac_11025	WP_002530685.1	Amino acid permease	Transport	Transporter activity	Membrane

upf. Universitat Pompeu Fabra Barcelona

Table S2. List of proteins identified exclusively in *C. acnes*-derived H1 EVs under anaerobic conditions

Gene name	Acession number	Protein name	GO - Biological process	GO - Molecular function	GO - Cellular component
HMPREF9578_00571	WP_002521853.1	Ribonuclease	Cellular homeostasis	Ribonuclease activity	Cytoplasm
pdxA cbac_01665	WP_002531202.1	4-hydroxythreonine-4-phosphate dehydrogenase PdxA	Biosynthetic process	Oxidoreductase activity	Cytoplasm
folE PPA0261 cbac_01480	WP_002517093.1	GTP cyclohydrolase 1	Metabolic process	Binding	Cytoplasm
PPA0503 cbac_02790	WP_002518798.1	Helix-turn-helix domain-containing protein	Regulation of biological process	Binding	Unknown
mmsA PPA0461 cbac_02540	WP_002531156.1	Methylmalonate-semialdehyde dehydrogenase	Metabolic process	Dehydrogenase activity	Cytoplasm
PPA1815 cbac_09865	WP_002517606.1	3-oxoacyl-ACP reductase FabG	Lipid metabolism	Reductase activity	Cytoplasm
PPA1609 cbac_08735	WP_002531040.1	Conserved phage-associated protein	Unknown	Unknown	Unknown
PPA2063 cbac_11190	WP_002530709.1	Peptide ABC transporter substrate-binding protein	Transport	Transporter activity	Membrane
PPA0851 cbac_04650	WP_002531018.1	ATP-dependent endonuclease	DNA repair	Hydrolase activity	Cytoplasm
PPA0144 cbac_00800	WP_011183610.1	DeoR/GlpR transcriptional regulator	Transcription	Molecular function regulator	Nucleoid
PPA1638 cbac_08890	WP_002531028.1	ABC transporter	Transport	Transporter activity	Membrane
PPA2091 cbac_11310	WP_002530732.1	ABC transporter ATP-binding protein	Transport	Transporter activity	Membrane
PPA1760 cbac_09575	WP_002514763.1	ABC transporter associated permease	Transport	Transporter activity	Membrane
PPA0399 cbac_02205	WP_002531171.1	Sugar ABC transporter	Transport	Transporter activity	Membrane
PPA1147 cbac_06215	WP_002513567.1	PTS sugar transporter subunit IIB	Transport	Kinase activity	Membrane
PPA1915 cbac_10385	WP_002518040.1	ABC transporter ATP-binding protein	Energy process	Binding	Membrane
cbac_02210	WP_002517551.1	Carbohydrate ABC transporter permease	Transport	Transporter activity	Membrane
PPA0925 cbac_05045	WP_002513798.1	Putative two-component system sensor kinase	Response to stimulus	Kinase activity	Membrane
PPA1599 cbac_08685	WP_007400937.1	CAAX protease	Protein processing	Peptidase activity	Membrane
PPA1389 cbac_07600	WP_002516495.1	NUDIX hydrolase	Cellular homeostasis	Hydrolase activity	Cytoplasm
PPA1721 cbac_09365	WP_002522139.1	Dihydrolipoamide dehydrogenase	Cellular homeostasis	Dehydrogenase activity	Cytoplasm
iolB PPA0458 cbac_02525	WP_002531157.1	5-deoxy-glucuronate isomerase	Metabolic process	Isomerase activity	Cytoplasm
PPA0398 cbac_02200	WP_002520024.1	Conserved protein, putative sugar-binding protein	Regulation of biological process	Binding	Membrane
cbac_10405	WP_002519594.1	CoA ester lyase	Unknown	Lyase activity	Unknown
PPA1529 cbac_08340	WP_002516971.1	Conserved protein	Unknown	Unknown	Membrane
moaC PPA0498 cbac_02765	WP_007401059.1	Cyclic pyranopterin monophosphate synthase	Biosynthetic process	Synthase activity	Cytoplasm
menB PPA0907 cbac_04940	WP_002513816.1	1,4-dihydroxy-2-naphthoyl-CoA synthase	Biosynthetic process	Synthase activity	Cytoplasm
PPA1229 cbac_06650	WP_002517946.1	ArsR family transcriptional regulator	Transcription	Molecular function regulator	Cytoplasm
PPA1086 cbac_05900	WP_002517877.1	Bifunctional nuclease family protein	Protein processing	Hydrolase activity	Nucleoid
PPA2112 cbac_11430	WP_002530753.1	DNA-binding response regulator	Transcription	Binding	Nucleoid
PPA1459 cbac_07975	WP_002514269.1	Alpha-mannosidase	Metabolic process	Binding	Cytoplasm
PPA1050 cbac_05695	WP_011183787.1	Conserved protein	Unknown	Unknown	Unknown
PPA0062 cbac_00345	WP_002530850.1	Alpha-mannosidase	Metabolic process	Binding	Cytoplasm
aceE cbac_05385	WP_002525003.1	Pyruvate dehydrogenase E1 component	Metabolic process	Dehydrogenase activity	Cytoplasm
PPA2267 cbac_12320	WP_002519750.1	6-phosphofructokinase	Metabolic process	Kinase activity	Cytoplasm
topA PPA0241 cbac_01375	WP_002529762.1	DNA topoisomerase 1	DNA replication	Binding	Nucleoid
nadE PPA2266 cbac_12310	WP_002518158.1	Glutamine-dependent NAD	Biosynthetic process	Synthase activity	Cytoplasm
PPA1142 cbac_06190	WP_002521618.1	PTS system, sucrose-specific IIBC component	Transport	Transferase activity	Membrane
cbac_04110	WP_002530961.1	DUF3040 domain-containing protein	Unknown	Unknown	Membrane
aroC cbac_06400	WP_002517990.1	Chorismate synthase	Biosynthetic process	Synthase activity	Cytoplasm

cbac_10960	WP_002519475.1	MFS transporter	Transport	Transporter activity	Membrane
gnpA PPA0083 cbac_00455	WP_002530846.1	1,3-beta-galactosyl-N-acetylhexosamine phosphorylase	Metabolic process	Phosphorylase activity	Cytoplasm
PPA2320 cbac_12625	WP_002517435.1	L-arabinose utilization protein, glycerol dehydrogenase	Lipid metabolism	Oxidoreductase activity	Cytoplasm
PPA0290 cbac_01620	WP_002517197.1	Putative two-component sensor kinase	Response to stimulus	Structural molecular activity	Membrane
ctaD PPA0702 cbac_03885	WP_002518277.1	Cytochrome c oxidase subunit 1	Transport	Binding	Membrane
PPA2203 cbac_11960	WP_002518166.1	Putative Na+/H+ antiporter	Transport	Transporter activity	Membrane
PPA1315 cbac_07150	WP_009640225.1	Exodeoxyribonuclease V gamma chain	DNA repair	Ribonuclease activity	Cytoplasm
cbac_10755	WP_002523368.1	Pyridine nucleotide-disulfide oxidoreductase	Cellular homeostasis	Oxidoreductase activity	Nucleoid
PPA1610 cbac_08740	WP_002531039.1	Putative transcriptional regulator	Transcription	Binding	Nucleoid
PPA0416 cbac_02280	WP_011183669.1	DNA-binding response regulator	Transcription	Binding	Nucleoid
PPA2216 cbac_12040	WP_002514161.1	Acyl-CoA dehydrogenase	Lipid metabolism	Dehydrogenase activity	Cytoplasm
PPA1273 cbac_06890	WP_002516865.1	ABC transporter ATP-binding protein	Transport	Transporter activity	Membrane
cbac_02265	WP_002517453.1	ABC transporter ATP-binding protein	Transport	Transporter activity	Membrane
cobA PPA0439 cbac_02395	WP_002517252.1	Uroporphyrinogen III methylase	Biosynthetic process	Transferase activity	Cytoplasm
argC HMPREF0675_4395	WP_002520173.1	N-acetyl-gamma-glutamyl-phosphate reductase	Biosynthetic process	Reductase activity	Cytoplasm
PPA2088 cbac_11295	WP_002530729.1	Esc family protein	Unknown	Unknown	Membrane
rsmH cbac_04135	WP_002530963.1	Ribosomal RNA small subunit methyltransferase H	Translation	Transferase activity	Cytoplasm
PPA1217 cbac_06590	WP_002518927.1	4-hydroxybutyrate coenzyme A transferase	Metabolic process	Transferase activity	Cytoplasm
PPA0141 cbac_00780	WP_002530823.1	HPr family phosphocarrier protein	Transport	Transferase activity	Cytoplasm
PPA2240 cbac_12185	WP_002530896.1	Cation-transporting ATPase	Transport	Transporter activity	Membrane
HMPREF0675_5099	WP_002516194.1	TrkA C-terminal domain protein	Transport	Transporter activity	Membrane
PPA1732 cbac_09420	WP_002531250.1	ABC transporter associated permease	Transport	Transporter activity	Membrane
PPA0540 cbac_03005	WP_002516643.1	Conserved protein // PrsW family intermembrane metalloprotease	Regulation of biological process	Peptidase activity	Membrane
PPA0068 cbac_00375	WP_002519827.1	Putative two component sensor kinase	Response to stimulus	Kinase activity	Membrane
PPA1275 cbac_06900	WP_002516777.1	ABC transporter permease	Transport	Transporter activity	Membrane

Universitat Pompeu Fabra Barcelona

Table S3. List of proteins identified exclusively in *C. acnes*-derived H2 EVs under anaerobic conditions

Gene name	Accession number	Protein name	GO - Biological Process	GO - Molecular function	GO - Cellular component
PPA1345 cbac_07350	WP_002517702.1	DUF1707 domain-containing protein	Unknown	Unknown	Membrane
HMPREF0675_3894	WP_002515660.1	Uncharacterized protein	Unknown	Unknown	Membrane
rpsT HMPREF0675_3956	WP_002515457.1	30S ribosomal protein S20	Translation	Structural molecular activity	Ribosome
PPA0522 cbac_02900	WP_011183691.1	Nitrate reductase	Energy process	Transporter activity	Membrane
PPA0960 cbac_05215	WP_002517830.1	MsnO8 family LLM class oxidoreductase	Regulation of biological process	Oxidoreductase activity	Membrane
PPA0960 cbac_05215	WP_002517830.1	MsnO8 family LLM class oxidoreductase	Response to stimulus	Oxidoreductase activity	Cytoplasm
PPA0724 cbac_04005	WP_002530950.1	1-acyl-sn-glycerol-3-phosphate acyltransferase	Lipid metabolism	Transferase activity	Cytoplasm
hisB HMPREF9578_00150	WP_002513559.1	Imidazoleglycerol-phosphate dehydratase	Byosynthetic process	Lyase activity	Cytoplasm
kup cbac_11865	WP_002519550.1	Probable potassium transport system protein kup	Transport	Transporter activity	Membrane
uppP HMPREF0675_5297	WP_002516473.1	Undecaprenyl-diphosphatase	Cell wall organization and cell shape	Phosphatase activity	Membrane
PPA0619 cbac_03440	WP_002530923.1	Allantoin permease	Transport	Transporter activity	Membrane

Table S4. List of primer sequences used for RT-qPCR

Genes		Primers (From 5' to 3')
CREBBP	FWD	5' GAGAGCAAGCAAACGGAGAG
	RV	5' AAGGGAGGCAAACAGGACA
TNF*a* SEB	FWD	5' CCAGGGACCTCTCTCTAATCA
	RV	5' TCAGCTTGAGGGTTTGCTAC
IL-6 SEB	FWD	5' ACTCACCTCTTCAGAACGAATTG
	RV	5' AGCCATCTTTGGAAGGTTCAG
IL-8 SEB	FWD	5' CTTGGCAGCCTTCCTGATTT
	RV	5' GGGTGGAAAGGTTTGGAGTATG
PLIN 2	FWD	5' TCAGCTCCATTCTACTGTTCACC
	RV	5' CCTGAATTTTCTGATTGGCACT
TGFβ1	FWD	5' TACCTGAACCCGTGTTGCTCTC
	RV	5' GTTGCTGAGGTATCGCCAGGAA
COX-2	FWD	5' GAATCATTCACCAGGCAAATTG
	RV	5' TCTGTACTGCGGGTGGAACA
OCLN	FWD	5' GTCATCCAGGCCTCTTGAAA
	RV	5' GGTGATAATGATTCGGTTTG
MMP2	FWD	5' AGCGAGTGGATGCCGCCTT
	RV	5' CATTCCAGGCATCTGCGAT
CLDN1	FWD	5' GTCTTTGACTCCTTGCTGAATCTG
	RV	5' CACCTCATCGTCTTCCAAGCAC
IL-10	FWD	5' TCTCCGAGATGCCTTCAGCAGA
	RV	5' TCAGACAAGGCTTGGCAACCCA

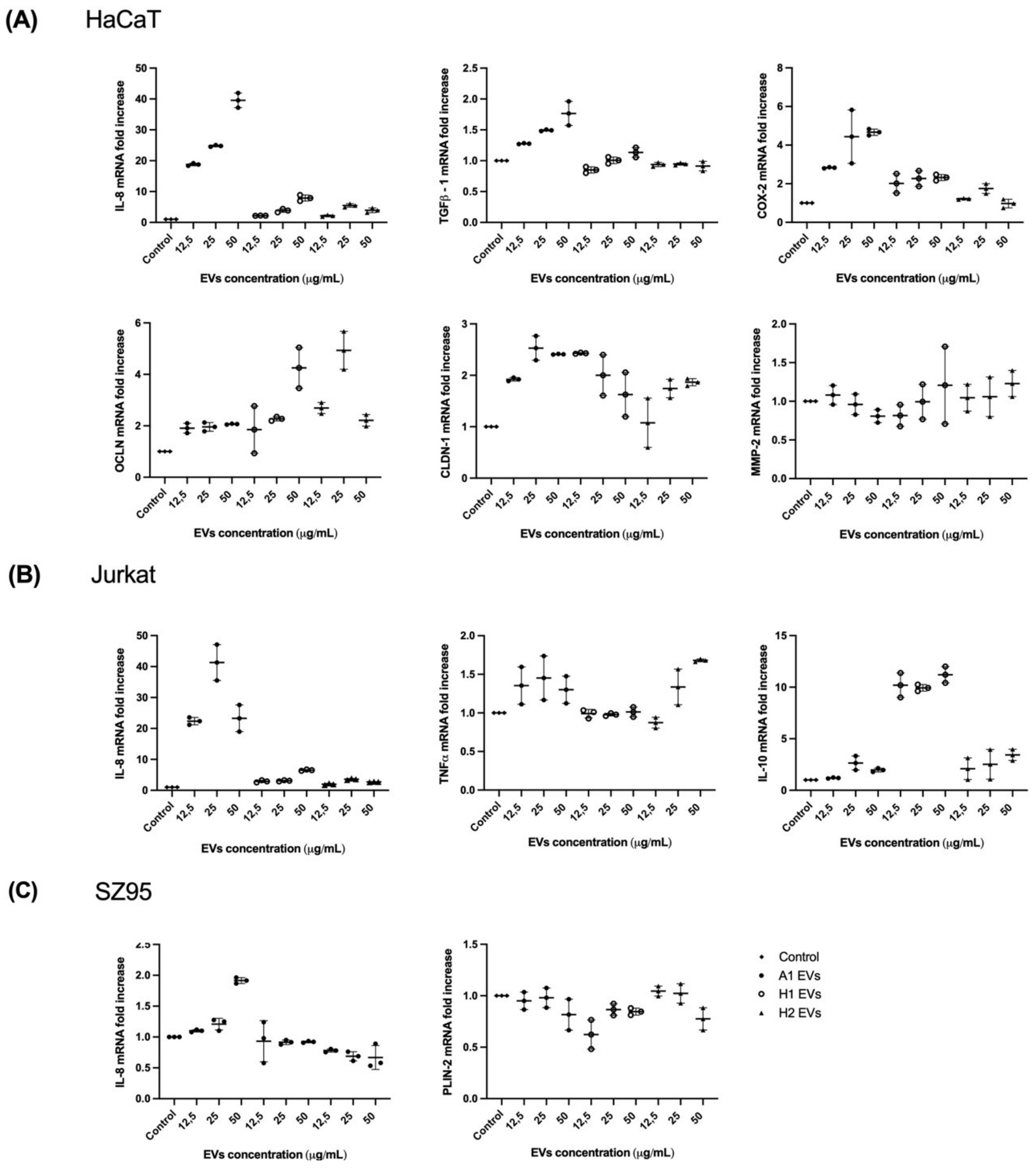

Figure S1. *In vitro* analysis with direct incubation of *C. acnes* EVs. Human cells were treated for 24h with different concentrations (12.5, 25 and 50 µg/mL) of *C. acnes* A1, H1 and H2 EVs. Total RNA was extracted, and different biomarkers were assessed by RT-qPCR. (A) HaCaT (B) SZ95 and (C) Jurkat immortalized cell lines. All data are presented as individual dots of experimental replicates ± standard deviation (SD) of triplicate measurements (*p ≤ 0.05, **p ≤ 0.001 versus non-stimulated controls).

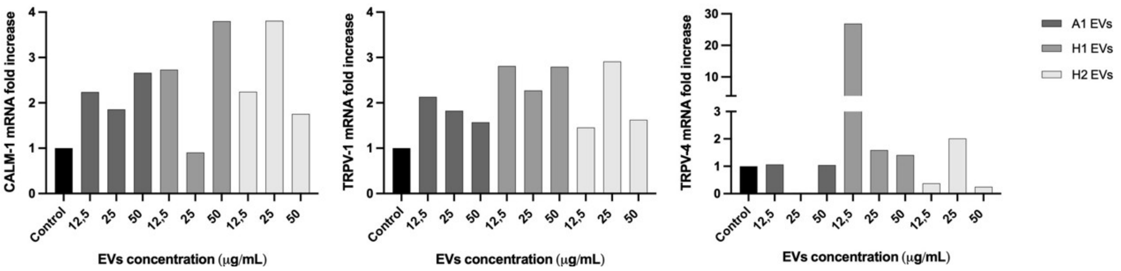

Figure S2. *In vitro* analysis with direct incubation of *C. acnes* EVs with CALM-1, TRPV-1, and TRPV-4 biomarkers. HaCaT cells were treated for 24h with different concentrations (12.5, 25 and 50 µg/mL) of *C. acnes* A1, H1 and H2 EVs. Total RNA was extracted, and different biomarkers were assessed by RT-qPCR.

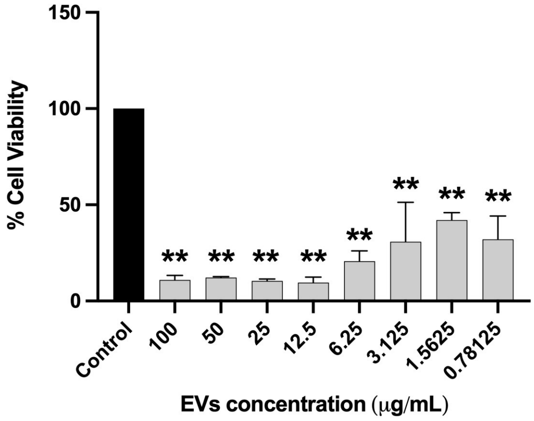

Figure S3. Cell viability assay of HaCaT cells incubated with ExoGAG. Different concentrations of pure ExoGAG (IDIS Nasasbiotech) were tested in an *in vitro* skin model of HaCaT cells to study the tolerance of this product as an alternative method to precipitate and isolate EVs. All data are presented as mean ± standard deviation (SD) of triplicate measurements (**$p \leq 0.001$ versus non-stimulated controls).

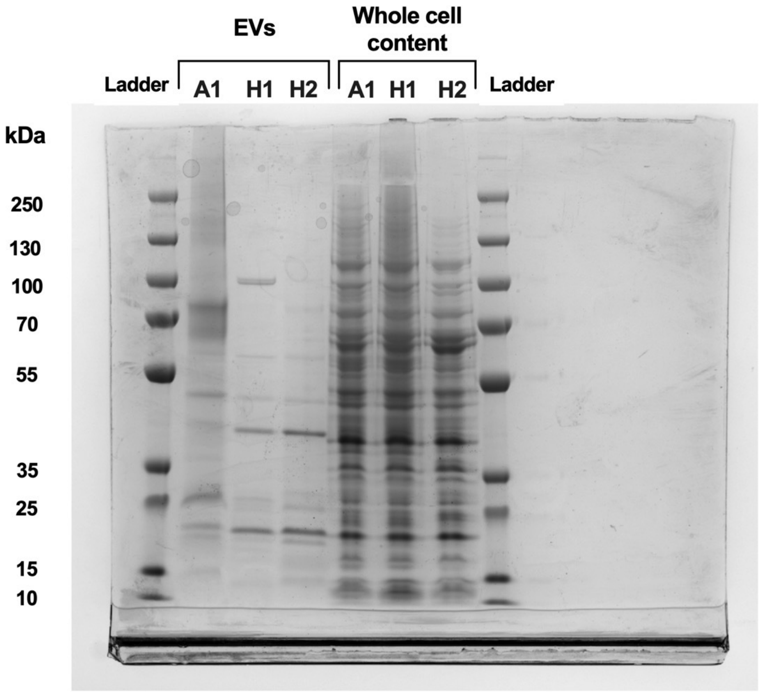

Figure S4. SDS Gel. Protein profile of *C. acnes* A1, H1 and H2. Both EVs and whole cell content fractions are represented.
